# The Th-Acetate Chemical Equilibria: Is It Really That Simple?

**DOI:** 10.1021/acs.inorgchem.5c03418

**Published:** 2025-11-10

**Authors:** Janik Lohmann, Christelle Tamain, Philippe Moisy, Tobias Reich, Jean Aupiais

**Affiliations:** 1 Department of Chemistry-Nuclear Chemistry, Johannes Gutenberg-Universität Mainz, Mainz 55099, Germany; 2 CEA, DES, ISEC, DMRC, Univ Montpellier, Marcoule F-30207, France; 3 CEA, DAM, DIF, Arpajon Cedex F-91297, France

## Abstract

The Th/acetate chemical system is truly unique. While it was generally accepted that complexes with one to five acetate ligands are formed, in this work, the formation of hydrolyzed thorium acetate species has been discovered, leading to the redefinition and revisiting of this system. Using the coupling between capillary electrophoresis and ICP-MS, the first four Th-AcO constants have been re-evaluated. Under the experimental conditions, [Th­(AcO)_5_]^−^ was not observed. Instead, [Th­(OH)­(AcO)*_i_*]^3–*i*^(*i* = 3,4) species were detected. In CE-ICP-MS, kinetically stable species are studied by evaluating the peak areas, while labile species are studied through variations in electrophoretic mobility. For Th, both types of complexes have been observed simultaneously. Based on the variations in the peak area, we were able to determine the first Th^4+^ hydrolysis constant (log*K ^0^ = −2.7 ± 0.2), in agreement with the value recommended by the Nuclear Energy Agency (NEA) (log*K ^0^ = −2.5 ± 0.5). Variations in electrophoretic mobility enabled us to determine the constants β_*i*_ (*i* = 1 – 4) of [Th­(AcO)*_i_*]^4–*i*^ complexes at two ionic strengths (0.1 and 0.3 M NaClO_4_) and to extrapolate them with other data found in literature to zero ionic strength using the specific ion interaction theory (SIT). By combining the constants for the hydrolysis and the binary Th-AcO complexes, the formation constants for the [Th­(OH)­(AcO)_*i*_]^3–*i*^ (*i* = 1 – 4) species were calculated.

## Graphical abstract

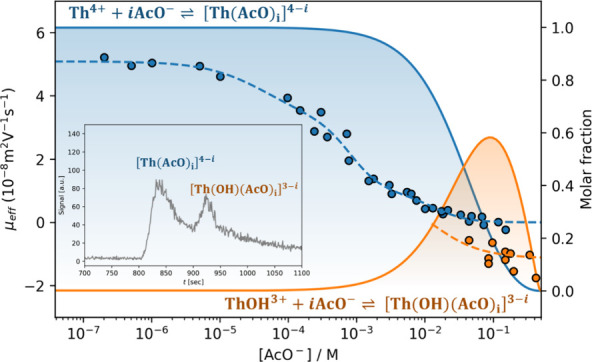

## 1 Introduction

Acetic acid (AcOH) is one of the simplest organic acids found in the environment. Although the literature is abundant with most metals, literature relating to the complexation of tetravalent actinides with this ligand is sparse.^1–4^ As a result, there are no selected data in the NIST database for the cations U^4+^, Pu^4+^, and Np^4+^.^5^ However, this is not the case for Th^4+^, where several references are given.^4,6–10^ The first aim of this work was to provide a reliable thermodynamic data set for tetravalent plutonium using a technique that allows performing speciation at ultra trace scale: capillary electrophoresis coupled to an ICP mass spectrometer (CE-ICP-MS). However, in order to guarantee the reliability of the results, we have included Th^4+^ in the same solution for comparison with the existing literature. The surprising results obtained for Th­(IV) led us to discuss and publish these results separately in this article, related to thorium only. The results for plutonium will be the subject of a separate forthcoming article.

There are some data available concerning the complexation of thorium by the acetate anion (cf. Table 1). In the 1970s Portanova et al. investigated the Th-AcO complexation for thorium concentrations ranging from 5 to 40 mM and in 1 molal sodium perchlorate medium using potentiometry.^7,8^ The obtained complex formation constants were consistent. In 2004, Rao et al. used the same technique at thorium concentrations, probably of the same order (mM) but not mentioned.^9^ These latest results seem to confirm earlier findings. However, in 1999, Moore et al. determined the successive constants by another technique, liquid–liquid extraction, and by measuring the alpha activity of the ^230^Th isotope by liquid scintillation.^4^ The experiments were carried out at various ionic strengths from 0.3 to 5 molals in sodium chloride medium. In this study, thorium was therefore at very low concentrations, probably in the nanomolar range. The results, extrapolated to zero ionic strength using the Specific Ion Interaction Theory (SIT), are in agreement with those obtained previously by potentiometry, also extrapolated to zero ionic strength. Recently, Willberger et al. have determined a new data set for the successive 1:1 to 1:5 complexes, using a technique very different from the previous ones – CE-ICP-MS. The determined constants are also in agreement with previous literature.^10^ Therefore, it seems that all the data currently available are consistent and that the Th-AcO system can be considered as well-known.

**Table 1:** Complexation Constants for the Th-AcO System in Different Media and at Different Temperatures^a^

medium, *I*, *θ* (°C)	method	species	log β_*i*_^*I*^	ref.
KNO_3_, 0.5 M, 25	potentiometry	[Th(AcO)]^3+^	3.12	^6^
		[Th(AcO)_2_]^2+^	6.29	
NaClO_4_, 1 M, 20	potentiometry	[Th(AcO)]^3+^	3.88 ± 0.02	^8^
		[Th(AcO)_2_]^2+^	6.91 ± 0.03	
		[Th(AcO)_3_]^+^	9.05 ± 0.05	
NaClO_4_, 1 M, 25	potentiometry	[Th(AcO)]^3+^	3.86 ± 0.02	^7^
		[Th(AcO)_2_]^2+^	6.97 ± 0.02	
		[Th(AcO)_3_]^+^	8.94 ± 0.03	
		[Th(AcO)_4_]_(**aq**)_	10.28 ± 0.06	
		[Th(AcO)_5_]^−^	11.00 ± 0.07	
NaClO_4_, 1 M, 25	potentiometry	[Th(AcO)]^3+^	3.79 ± 0.02	^9^
		[Th(AcO)_2_]^2+^	6.79 ± 0.02	
		[Th(AcO)_3_]^+^	8.71 ± 0.13	
		[Th(AcO)_4_]_(**aq**)_	10.17 ± 0.25	
		[Th(AcO)_5_]^−^	11.41 ± 0.22	
NaCl, 0.3 m, 25	solvent extraction	[Th(AcO)]^3+^	3.73 ± 0.02	^4^
		[Th(AcO)_2_]^2+^	7.47 ± 0.03	
NaCl, 1.0 m, 25	solvent extraction	[Th(AcO)]^3+^	3.85 ± 0.02	^4^
		[Th(AcO)_2_]^2+^	6.56 ± 0.03	
NaCl, 2.0 m, 25	solvent extraction	[Th(AcO)]^3+^	3.92 ± 0.03	^4^
		[Th(AcO)_2_]^2+^	6.82 ± 0.03	
NaCl, 3.0 m, 25	solvent extraction	[Th(AcO)]^3+^	4.26 ± 0.03	^4^
		[Th(AcO)_2_]^2+^	7.19 ± 0.03	
NaCl, 4.0 m, 25	solvent extraction	[Th(AcO)]^3+^	4.29 ± 0.03	^4^
		[Th(AcO)_2_]^2+^	7.30 ± 0.03	
NaCl, 5.0 m, 25	solvent extraction	[Th(AcO)]^3+^	4.51 ± 0.03	^4^
		[Th(AcO)_2_]^2+^	7.66 ± 0.03	
NaCl, 0.3–5 m, 25, extrapolation to *I* = 0 using SIT		[Th(AcO)]^3+^	5.24	^4^
		[Th(AcO)_2_]^2+^	9.06	
NaClO_4_, 0.3 M, 25	CE-ICP-MS	[Th(AcO)]^3+^	3.66 ± 0.16	^10^
		[Th(AcO)_2_]^2+^	7.04 ± 0.09	
		[Th(AcO)_3_]^+^	9.75 ± 0.11	
		[Th(AcO)_4_]_(**aq**)_	10.28 ± 0.87	
		[Th(AcO)_5_]^−^	11.75 ± 0.16	
NaClO_4_, 0.3 M, 25	CE-ICP-MS	[Th(AcO)]^3+^	4.00 ± 0.23	merged data: this study + raw data in Willberger et al.^10^
		[Th(AcO)_2_]^2+^	7.12 ± 0.30	
		[Th(AcO)_3_]^+^	10.10 ± 0.22	
		[Th(AcO)_4_]_(**aq**)_	11.9 ± 0.6	
NaClO_4_, 0.1 M, 25	CE-ICP-MS	[Th(AcO)]^3+^	4.18 ± 0.20	this study
		[Th(AcO)_2_]^2+^	7.46 ± 0.16	
		[Th(AcO)_3_]^+^	9.60 ± 0.17	
		[Th(AcO)_4_]_(**aq**)_	11.18 ± 0.12	

a Constants are expressed in the unit of the medium.

Nevertheless, the case of thorium is not as simple as it seems. In this article, we show that for Th^4+^ the stability constants related to the 1:4 and 1:5 complexes published in literature are probably biased due to the presence of Th-OH-AcO species at pH > 3. The negatively charged [Th(OH)(AcO)_4_]^−^ complex was most likely also present in previous experiments but was interpreted as [Th­(AcO)_5_]^−^. The assumption of the latter complex influences the determination of the preceding [Th­(AcO)_4_]_(aq)_ complex. In practice, thanks to the ability of CE-ICP-MS to separate chemical species based on their charge, we have discovered the existence of Th-OH-AcO species. This calls in question the literature values found for the 1:4 and 1:5 complexes. These Th-OH-AcO species were previously detected by Willberger et al., but unfortunately not interpretated or considered as actual Th species.^10^ We have therefore prepared new experiments which, when compared with the previous ones,^10^ extend the acetate concentration to better observe all species (especially the 1:1 complex). In turn, the data collected by Willberger et al.^10^ have been reprocessed regarding the Th-OH-AcO species.

## 2 Material and Methods

All solutions used in this study were prepared daily. All reagents used were of analytical grade and were characterized as being useable for trace analyses.

Safety Precaution: **Caution!** Thorium and plutonium are radioactive, alpha-emitting elements that must be handled in an appropriate laboratory and require special caution and radiation protection.

### 2.1 Sample Preparation in 0.3 M NaClO_4_

The background electrolyte (BGE) solutions were prepared by varying the pH value of a 0.5 M acetic acid solution (Fisher Scientific GmbH, Schwerte, Germany) using 1 M HClO_4_ (Sigma-Aldrich, St. Louis, Missouri, USA) and 1 M NaOH (VWR, Darmstadt, Germany). Each sample was prepared by adding various volumes of acid or base. For each BGE, the ionic strength was adjusted to 0.3 M using 2 M NaClO_4_ solution (prepared by dissolution of NaClO_4_ solid from Sigma-Aldrich, St. Louis, Missouri, USA, analytical grade 99.5%). The volume was adjusted to 1 mL using deionized water (Milli-Q 18 MΩ, Millipore, Burlington, Massachusetts, USA). The details of the electrolyte preparations are given in Table S3 in the Supporting Information (SI).

The pH values were measured using the high-precision 780 pH meter (Metrohm, Herisau, Switzerland) equipped with a Metrohm LL combined pH glass electrode. To prevent the appearance of a junction potential, the 3 M KCl electrolyte of the pH electrode was replaced by a solution of 0.3 M NaClO_4_. In addition, pH solutions were prepared from standardized stock solutions of 0.97 M HClO_4_ and 2 M NaClO_4_. Characteristics of the solutions and performance of the pH electrode are reported in Table S7 and Figure S2, Supporting Information.

Each actinide sample was prepared by adding various volumes of 1 M (or 0.1 M) NaOH, 1 M (or 0.1 M) HClO_4_, 2 M NaClO_4_, 1 M acetic acid, and actinide stock solutions. The plutonium stock solution was ^239^Pu­(IV) in chloride media. The concentration of the ^232^Th­(IV) stock solution was 2 × 10^–6^ M. Plutonium and thorium solutions were prepared by evaporation and redissolution in 0.5 M acetic acid to form 1 × 10^–7^ M and 2 × 10^–6^ M solutions, respectively. The presence of both actinides in solution does not modify the behavior of the systems separately, given the large excess of acetate in the solutions. Then, 5 μL of each actinide solution were mixed with various volumes of 1 M (or 0.1 M) NaOH, 1 M (or 0.1 M) HClO_4_, 2 M NaClO_4_, and 1 M acetic acid to form a 100 μL sample with the same characteristics as the BGE besides the presence of thorium and plutonium with a final concentration of about 10^–7^ M and 5 × 10^–9^ M, respectively. The complex [GaNOTA] (NOTA = 1,4,7-Triazacyclononane-1,4,7-triacetic acid) was used as a neutral marker in these analyses. [GaNOTA] was prepared using a standard solution of gallium (1000 μg·mL^–1^, Spex Certiprep, Stanmore, UK) and the molecule NOTA at 14 mmol·L^–1^ (Chematech, Dijon, France) with an excess of gallium to ensure that there was no trace of free NOTA. The details of the sample preparations are given in Table S2, Supporting Information.

The separations were performed using a fused-silica capillary with 50 μm internal diameter and 77.5 cm length at +4 kV for pH ≤ 0.6, and ± 7 kV for pH > 0.6. The voltage was chosen so that the temperature increase inside the capillary does not exceed 1 °C. The setup consists of a Sciex PA 800 Plus capillary electrophoresis (Sciex, Framingham, Massachusetts, USA) maintained at 25 °C during the analysis, a MiraMist CE Nebulizer (Burgener Research, Mississauga, Canada), and an ICP-MS Agilent 8900 QQQ (Agilent, Santa Clara, California, USA).

### 2.2 Sample Preparation in 0.1 M NaClO_4_

The background electrolytes (BGE) were produced by varying the pH value of a 0.75 M acetic acid solution (Fluka, Buchs, Switzerland) using 9 M HClO_4_ (VWR, Darmstadt, Germany) and 10 M NaOH (Merck, Darmstadt, Germany). For each BGE, the ionic strength was adjusted to 0.1 M using NaClO_4_ (Merck, Darmstadt, Germany). To produce samples with a lower AcO^–^ concentration, some BGEs were diluted accordingly in NaClO_4_/HClO_4_ solution of pH 1.3 and *I* = 0.1 M.

The pH meter inoLab pH 720 (Xylem, Weilheim, Germany) equipped with a BlueLine 16 pH microelectrode (SI Analytics, Mainz, Germany, 3 M NaCl) were used for pH measurements. As the commercially available pH buffers used for the calibration were also at an ionic strength of 0.1 M, no correction for ionic strength was performed.

The 2 × 10^–4^ M ^239^Pu­(IV) stock solution was prepared by electrolysis in 1 M HClO_4_. A detailed description of the preparation is given in Stietz et al.^11^ The oxidation state was confirmed by UV–vis spectroscopy (Tidas 100, J&M Analytik AG, Essingen, Germany, Figure S3, Supporting Information). For the ^232^Th­(IV) stock solution, an ICP-MS standard (Accu Trace, Accu Standard, New Haven, Connecticut, USA) of a known concentration was evaporated and redissolved in 0.1 M HClO_4_ to produce a 2 × 10^–4^ M Th­(IV) stock.

To 2 mL aliquots of the BGEs, 2 μL of each stock solution were added to produce the sample solutions with a final concentration of 2 × 10^–7^ M for each actinide. The details of the sample preparations are given in Table S4, Supporting Information.

For the CE measurements, 1 μL of 2-bromopropane (Merck, Darmstadt, Germany) was added to 200 μL of each actinide sample as a neutral marker. Capillary electrophoresis measurements were performed using an Agilent 7100 CE-System (Agilent, Santa Clara, California, USA) hyphenated to an Agilent 7900 ICP-MS (Agilent, Santa Clara, California, USA). This coupling was realized via a MiraMist CE Nebulizer (Burgener Research, Mississauga, Canada) and a Scott-type spray chamber (AHS Analysentechnik, Tübingen, Germany). The separations were performed at +10 kV using a fused-silica capillary with 50 μm internal diameter and 50 cm length. A pressure of 90 mbar was applied to aid the electroosmotic flow (EOF). The temperature was controlled at 25.0 ± 0.1 °C using the internal air cooling of the CE as well as a custom-built enclosure for the hyphenation.

### 2.3 Data Treatment of Thermodynamics

A fast chemical equilibrium between a cation and a ligand results in a single migration band containing all species of interest in equilibrium with each other. In this case, the key parameter is the effective electrophoretic mobility μ_eff_ and is determined using the following relationship:

$$\mu_{\mathrm{eff}}=\frac{\mu_{M}+\underset{i}{\sum }\beta_{i}\mu_{i}{\left[L\right]}^{i}}{1+\underset{i}{\sum }\beta_{i}{\left[L\right]}^{i}}$$ 1

where μ_M_ is the electrophoretic mobility of the uncomplexed cation, μ_*i*_ is the electrophoretic mobility of the complex *i*, β_*i*_ is the stability constant for the formation of complex *i* to be determined, and [L] is the free ligand concentration. Eq 1 is the outcome of successive equilibria between a metallic cation M and an anionic ligand L.

The effective electrophoretic mobility μ_eff_ can be calculated by eq 2 with the migration time of the actinide *t*_An_, the migration time of a neutral marker *t*_EOF_, indicating the EOF, the effective length *l* of the capillary, and the applied voltage *U.*

$$\mu_{\mathrm{eff}}=\frac{l^{2}}{U}\left(\frac{1}{t_{\mathrm{An}}}-\frac{1}{t_{\mathrm{EOF}}}\right)$$ 2

The electrophoretic mobilities for all measurements were calculated using eq 2 and are summarized in the Supporting Information (Tables S5 and S6).

The following procedure was applied for the extrapolation to zero ionic strength: All values obtained at a given ionic strength were first converted in molality when necessary; the NEA procedure was then applied to determine the values in the standard state and the SIT parameters.^12^ In the case where all ion interaction coefficients are known allowing a direct calculation at *I* = 0, then the uncertainty is calculated as follows: $\sigma_{\log_{10}\beta^{0}}=\sqrt{\sigma_{\log_{10}\beta }^{2}+{\left(m_{X^{-}}\sigma_{\Delta \epsilon }\right)}^{2}}$ , X = Cl^–^ or ClO_4_^–^. All uncertainties are given at 95% of confidence level. The ion interaction coefficients used in this work are summarized in Table S1, Supporting Information.

## 3 Results and Discussion

### 3.1 Identification of the Th-OH-AcO Species

A strange behavior was observed in the experiments at *I* = 0.3 M with increasing pH value and thus increasing acetate concentration. Only a single peak appears in the ^232^Th signal up to pH ≈ 3 (i.e., [AcO^–^] ≈ 20 mM). Above pH 3 two peaks are present in the electropherograms (see example Figure 1, left). Since the experiments at *I* = 0.1 M did not exceed pH 3.6 and had a higher acetate concentration, here only one peak of ^232^Th was observed per measurement.

**Figure 1:**
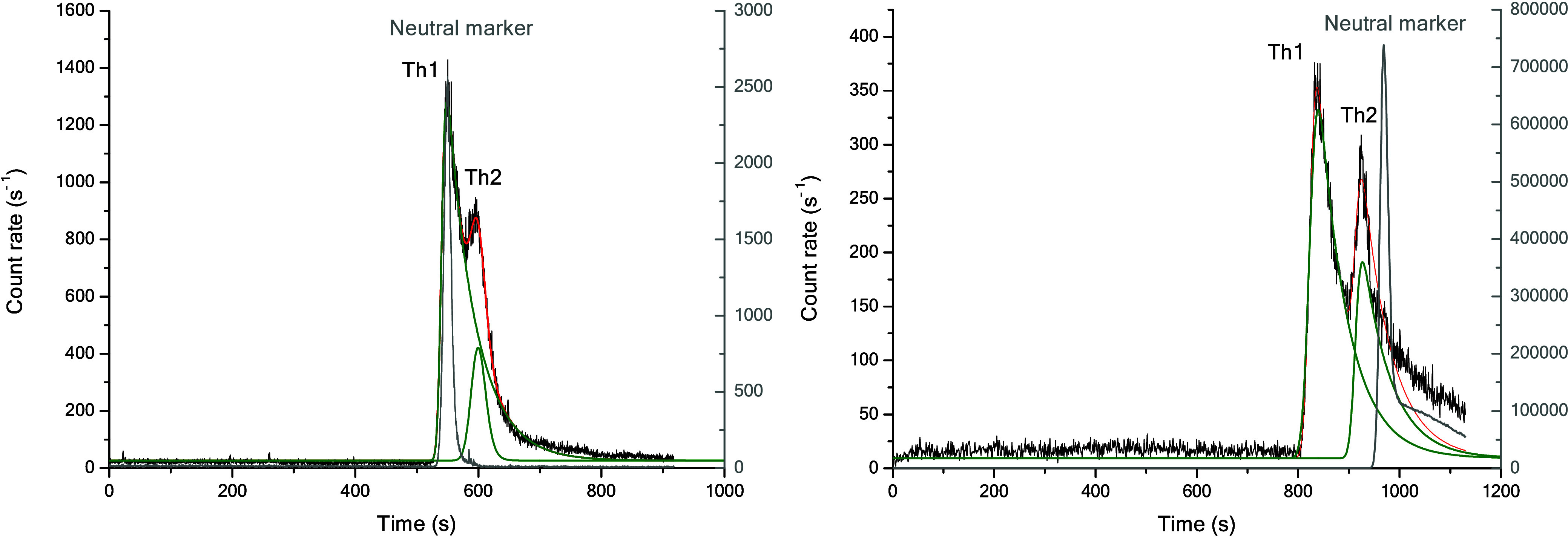
(left) Electropherogram of a solution of Th^4+^ (*c* = 2 × 10^–7^ M) in 0.3 M NaClO_4_, pH = 4.15, [AcO^–^] = 0.149 M, ^69^GaNOTA as neutral marker, *V* = +7 kV. (right) Electropherogram of a solution of Th^4+^ (*c* = 1 × 10^–6^ M) in 0.3 M NaClO_4_, pH = 3.39, [AcO^–^] = 0.0216 M, bromopropane (^79^Br) as neutral marker. The raw data from Willberger et al.^10^ were retreated by the authors, *V* = +10 kV. Th1 = Th-AcO system, Th2 = Th–OH-AcO system.

This unusual behavior also appeared in the previous experiments by Willberger et al. but was not investigated further (see example in Figure 1, right). At the time, only the peak labeled Th1 had been assigned to the acetate species.^10^ However, this evaluation is unsatisfactory, since the electropherograms were recorded for ^232^Th and thus all peaks should point back to Th species.

Despite the wide variation in acetate concentration between the two sets of experiments, the variation of the relative ratio between the two peaks is dependent only on pH (see Figure 2).

**Figure 2:**
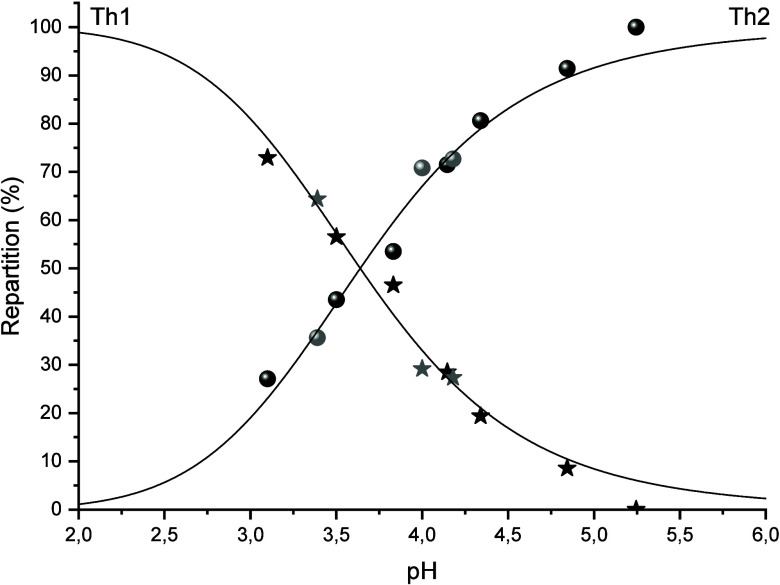
Variation of the relative areas of peaks Th1 (stars) and Th2 (sphere) as a function of the pH. In gray, retreatment of data from Willberger et al.^10^ by the authors, in black new experiments. The two curves intersect at pH = 3.64 ± 0.09.

This observation strongly suggests hydrolysis of the thorium acetate complexes. In fact, by taking into account the hydrolysis of Th^4+^ between pH 3 and 5 (see Figure S1, Supporting Information), under the experimental conditions Th­(IV) is mainly present as the first hydrolyzed complex Th­(OH)^3+^, interacting with acetate ligands. Therefore, we propose the following equilibrium:

$${\left[\mathrm{Th}{\left(\mathrm{AcO}\right)}_{i}\right]}^{4-i}+H_{2}O⇌{\left[\mathrm{Th}\left(\mathrm{OH}\right){\left(\mathrm{AcO}\right)}_{i}\right]}^{3-i}+H^{+}$$ 3

This reaction is not reversible in the time frame of a CE measurement as the two observed peaks are distinct and depend only on pH. The proposed reaction is independent of successive complexation reactions with acetate. Therefore, the species cannot be added to the overall equation for rapid equilibria and must be considered separately.^13^

In Figure 1 (left), the Th1 peak exhibits an electrophoretic mobility near zero, whereas peak Th2 exhibits a negative mobility value. In contrast both peaks in Figure 1 (right) exhibit a positive electrophoretic mobility in more acidic conditions at a lower acetate concentration. This result suggests that, in addition to an irreversible reaction, another reversible reaction takes place. It is worth noting that the variation of peak areas is related to the formation of kinetically stable complexes whereas the formation of labile complexes can be quantified by variations in electrophoretic mobility.^13^ Such behavior has never been observed before in CE-ICP-MS and requires treating the data in two steps. First, we will focus on peak areas to determine the formation constant of the hydrolysis reaction and second will treat the variation of electrophoretic mobilities to derive the stability constants relative to the formation of labile complexes.

### 3.2 Determination of the Hydrolysis Constant

The determination of the relative peak areas presented in Figure 2 allows the complexation constant of the hydrolysis reaction, log*K_OH,AcO_^0^, (eq 3) to be determined.

Indeed, at the equivalent point, were the concentrations of both hydrolyzed and nonhydrolyzed species are equal ([[Th­(AcO)_i_]^4–i^] = [[Th­(OH)­(AcO)_i_]^3–i^]), the stability constant log*K_OH,AcO_^0^ (eq 4) depends only on the H_(aq)_^+^ concentration (eq 5).

KOH,AcO*=[[Th(OH)(AcO)i]3−i]×[H+][[Th(AcO)i]4−i] 4

log⁡KOH,AcOI=0.3M*=log[H+]=−3.64±0.09 5

The extrapolation at zero ionic strength using SIT gives log*K_OH,AcO_^0^ = –2.73 ± 0.10. This result agrees with the value recommended in the NEA review by Rand et al.^14^ of log*K_OH_^0^ = –2.5 ± 0.5 for the hydrolysis of Th­(IV) in absence of acetate. At pH 3.6, about 90% of Th is shared between Th^4+^ and Th­(OH)^3+^. The contribution of Th­(OH)_2_^2+^ does not exceed 10%, a proportion hardly detectable in CE-ICP-MS. Considering the shape of Th peaks (see Figure 1), the peak of Th­(OH)_2_^2+^ is probably lost in the tailing of the Th­(OH)^3+^ peak due to its smaller mobility (charge +2 vs charge +3). In practice, the peak assigned to the species Th­(OH)^3+^ contains a variable proportion of the species Th­(OH)_2_^2+^ which depends on the pH. Therefore, such contribution must be subtracted. However, its content can only be calculated based on a theoretical repartition diagram based on recommended NEA stability constants. We have found that this contribution varies from 2% at pH 3 up to 15% at pH 3.8. We have determined that this bias decreases the value of log*K_OH,AcO_^0^ by a maximum of 0.1. It is therefore reasonable to increase the overall uncertainty to about 0.2. Finally, we propose the following value at zero ionic strength:

[Th(AcO)i]4−i+H2O⇌[Th(OH)(AcO)i]3−i+H+,log⁡KOH,AcO0*=−2.7±0.2 6

### 3.3 Determination of Complexation Constant for the Th-AcO System

After identification of the peaks corresponding to the Th-AcO system, the complexation constants can be re-evaluated. To determine the complexation constants for the Th-AcO system, the electrophoretic mobilities μ_*eff*_ assigned to the Th-AcO system were plotted as a function of the free acetate concentration [AcO^–^]_free_, which was calculated using eq 7 and the p*K*_*a*_^*I*^ value of AcOH (p*K*_*a*_^0^ = 4.756 ± 0.003 ^15^ extrapolated to the corresponding ionic strength (p*K*_*a*_^*I*=0.1M^ = 4.558 and p*K*_*a*_^*I*=0.3M^ = 4.517).

$${\left[{\mathrm{AcO}}^{-}\right]}_{\mathrm{free}}=\frac{c_{0}\times 10^{-pK_{a}}}{10^{-pK_{a}}+10^{-\mathrm{pH}}}$$ 7

The experimental data were fitted using eq 9 under the assumption of the following fast equilibria expressed by eq 8 (*i* = 1–5):

$${\mathrm{Th}}^{4+}+i{\mathrm{AcO}}^{-}⇌{\left[\mathrm{Th}{\left(\mathrm{AcO}\right)}_{i}\right]}^{4-i}$$ 8

$$\mu_{eff}=\frac{\mu_{0}+\overset{N}{\underset{i=1}{\sum }}\mu_{i}\beta_{i}{\left[\mathrm{Ac}O^{-}\right]}_{\mathrm{free}}^{i}}{1+\overset{N}{\underset{i=1}{\sum }}\beta_{i}{\left[\mathrm{Ac}O^{-}\right]}_{\mathrm{free}}^{i}}$$ 9

In eq 9, μ_0_ represents the individual electrophoretic mobility of Th^4+^ and μ_*i*_ those of the Th-AcO complexes. The exact procedure is described in detail in Willberger et al.^10^

### 3.4 *I* = 0.3 M NaClO_4_

The electrophoretic mobility is an intensive quantity. Two experiments, performed with the same electrolyte composition (concentration and pH), at the same temperature, regardless of the voltage used and the length of the capillary, must give the same result. Our previous experiments published in Willberger et al.^10^ were performed in 0.3 M NaClO_4_ and at a total concentration of 0.3 M acetic acid. We therefore carried out new experiments in the same electrolyte, at the same ionic strength, but chose to increase the total acetic acid concentration to 0.5 M to observe the higher complexes more easily. Thus, compared to the previous study,^10^ we have extended the concentration range of acetate to low values down to 10^–8^ M (10^–4^ M in previous study) and to high values up to 0.4 M (0.18 M in previous study). At low concentrations, this allowed us to reach the plateau of μ_eff_ and thus determine the start of the first complexation more precisely.

To determine the complex formation constants for the Th-AcO system, the raw data of this work and of Willberger et al.^10^ were merged. Only electrophoretic mobilities μ_*eff*_ assigned to the Th-AcO system (Th1 peaks) under previous consideration were used for the evaluation and plotted as a function of free acetate concentration [AcO^–^]_free_ in Figure 3 (black symbols).

**Figure 3:**
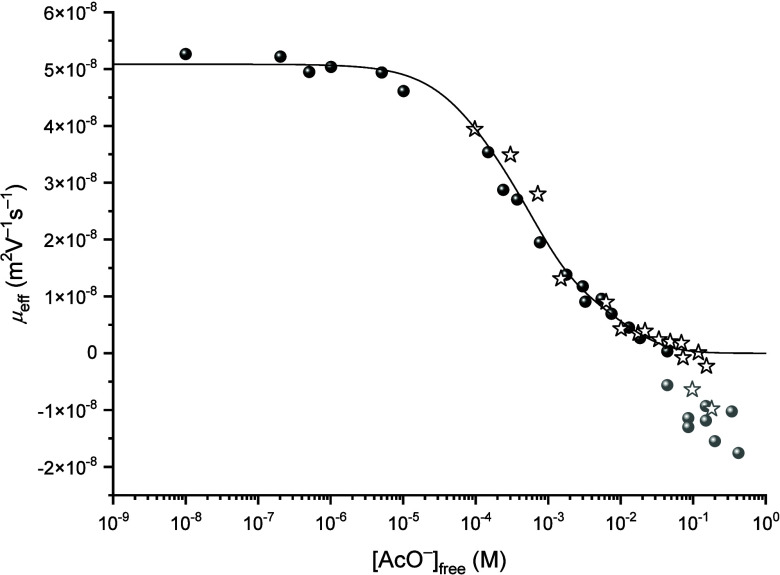
Variation of the effective mobility μ_*eff*_ of thorium as a function of the concentration of free acetate [AcO^–^]_free_ at *I* = 0.3 M, fitted using eq 9. Black sphere: new experiment, black star: retreated values by the authors from data in Willberger et al.^10^ Gray sphere and gray star: hydrolyzed thorium acetate complex, not considered in the fit.

The determined values of the complexation constants are gathered in Table 1, using the individual electrophoretic mobilities listed in Table 2. The trend in electrophoretic mobility shows a plateau at zero for high acetate concentrations. Interestingly, applying the same model as Willberger et al.,^10^ the mobility of the 1:5 complex was determined to be zero during the fitting procedure.

**Table 2:** Electrophoretic Mobility Values for the Species for the Th-AcO System Determined in this study at *I* = 0.3 M and *I* = 0.1 M

species	μ (10^–8^ m^2^ V^–1^ s^–1^) *I* = 0.3 M	μ (10^–8^ m^2^ V^–1^ s^–1^) *I* = 0.1 M
**Th** ^**4+**^	+5.086	+5.147
[**Th**(**AcO**)]**^3*+*^**	+3.243	+3.904
[**Th**(**AcO**)**_2_**]**^2*+*^**	+1.670	+2.169
[**Th**(**AcO**)**_3_**]**^*+*^**	+0.943	+1.090
[**Th**(**AcO**)**_4_**]	0	0

### 3.5 *I* = 0.1 M NaClO_4_

With the exception of one outlier, data procession was trouble-free as depicted in Figure 4.

**Figure 4:**
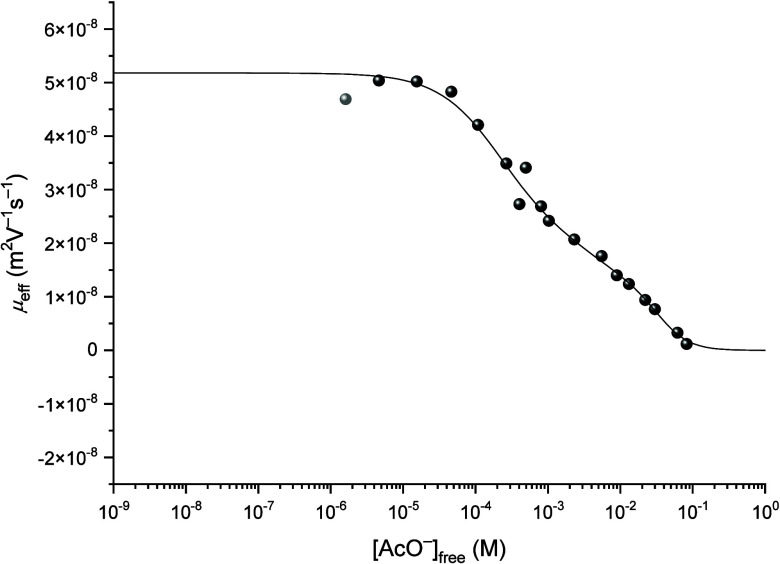
Variation of the effective mobility μ_*eff*_ of thorium as a function of the concentration of free acetate [AcO^–^]_free_ at *I* = 0.1 M, fitted using eq 9.

The complexation constants and mobilities are gathered in Tables 1 and 2, respectively. It is noted that for this set of data, the mobility of the potential 1:5 complex is again not negative, as expected, but very close to zero. The same was observed for experiments at 0.3 M NaClO_4_, which strongly suggests that the limiting Th-AcO complex under the experimental conditions is neutral. It is emphasized that this experimental result suggests three hypotheses:

1) The formation of a ternary complex of the type [NaTh­(AcO)_5_],
2) the protonation of the fifth acetate ligand [Th­(AcO)_4_(AcOH)] (similar to Am­(HDTPA)^−^),^16^
3) or more likely, only the complex [Th­(AcO)_4_] is detected as the limiting complex under the experimental parameters. At higher pH values and in turn higher free acetate concentration, the equilibrium shifts toward the hydrolyzed species described above.

### 3.6 Formation Constant of [Th­(AcO)]^3+^

Four values^7–9^ (including ours), obtained in NaClO_4_ medium at 25 °C are available in the literature (see Table 1). Thus, we have applied the NEA procedure^12^ to determine the stability constant at zero ionic strength for the formation of [Th­(AcO)]^3+^ (see Figure 5). We found: logβ_1_^0^ = 5.06 ± 0.11 and Δε = (–0.39 ± 0.14) kg mol^–1^ (Table 3). Using the known ion interaction coefficients summarized in Table S1, Supporting Information, the coefficient related to the species [Th­(AcO)]^3+^ was calculated to be ε_[Th(AcO)]^3+^,ClO_4_^–^_ = (0.39 ± 0.17) kg mol^–1^. We have retreated the data in 0.3–5 m NaCl medium by Moore et al.^4^ with the same procedure. It results in a slightly different value as published (see Table 1): logβ_1_^0^ = 5.07 ± 0.24 instead of 5.24. Both values are in agreement. The extrapolation at *I* = 0, using SIT parameters given in Table S1, Supporting Information from Sergeev et al.^6^ leads to a logβ_1_^0^ = 4.42. Due to the narrow range in [AcO^–^] from 5 × 10^–3^ M to 7.5 × 10^–2^ M investigated by Sergeev et al.,^6^ the complexation constant is most likely underestimated, as it is clear that Th-AcO complexes start forming at significantly lower [AcO^–^] of 1 × 10^–4^ M. Our final recommendation is reported in Table 3.

**Figure 5:**
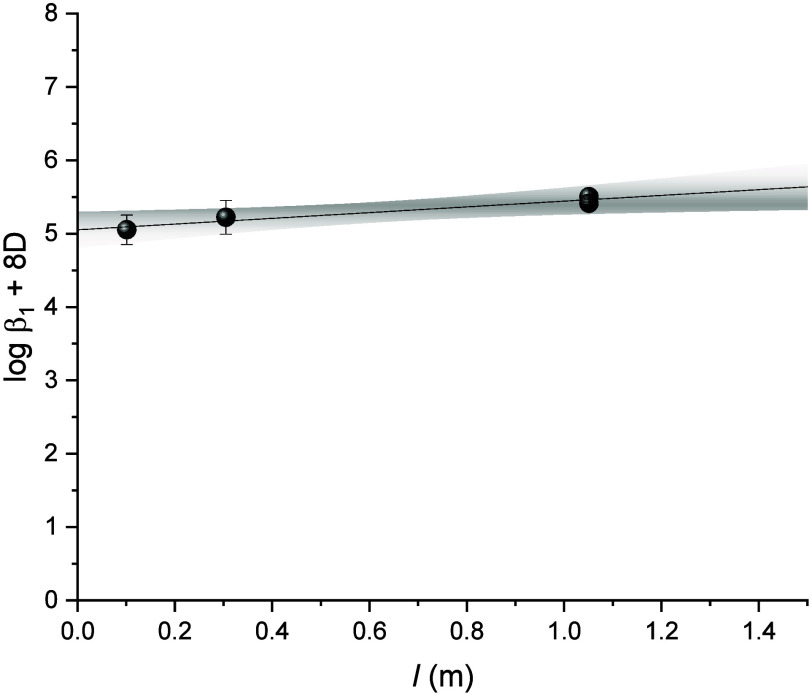
Extrapolation to *I* = 0 of experimental data for the formation of [Th­(AcO)]^3+^ using the specific interaction equation. The data are taken from references 7,9,10 and this study.

**Table 3:** Recommended Stability Constants at *I* = 0 for the Th-AcO System in NaClO_4_ Medium, 25 °C

equilibrium	log β_*i*_^0^
**Th** ^**4+**^ **+ AcO** ^**–**^ ⇌ [**Th**(**AcO**)]**^3*+*^**	5.06 ± 0.11
**Th** ^**4+**^ **+ 2AcO** ^**–**^ ⇌ [**Th**(**AcO**)**_2_**]**^2*+*^**	8.98 ± 0.20
**Th** ^**4+**^ **+ 3AcO** ^**–**^ ⇌ [**Th**(**AcO**)**_3_**]**^*+*^**	11.8 ± 0.5
**Th** ^**4+**^ **+ 4AcO** ^**–**^ ⇌ [**Th**(**AcO**)**_4_**]	13.9 ± 2.0

### 3.7 Formation Constant of [Th­(AcO)_2_]^2+^

From data of previous studies^7–9^ and this work, depicted in Figure 6, the following values have been obtained: logβ_2_^0^ = 8.98 ± 0.20 and Δε = (–0.71 ± 0.26) kg mol^–1^. It leads to a value for ε_[Th(AcO)_2_]^2+^,ClO_4_^–^_ = (0.15 ± 0.10) kg mol^–1^. The original data in 0.3–5 m NaCl medium by Moore et al.^4^ have been reprocessed with the same procedure. It gives logβ_2_^0^ = 9.28 ± 0.28 which agrees with our calculation in NaClO_4_ medium. Concerning the value proposed by Sergeev et al.,^6^ the extrapolation at *I* = 0, using SIT parameters given in Table S1 gives a smaller value of logβ_2_^0^ = 8.58, which is most likely also underestimated. The final recommendation is reported in Table 3.

**Figure 6:**
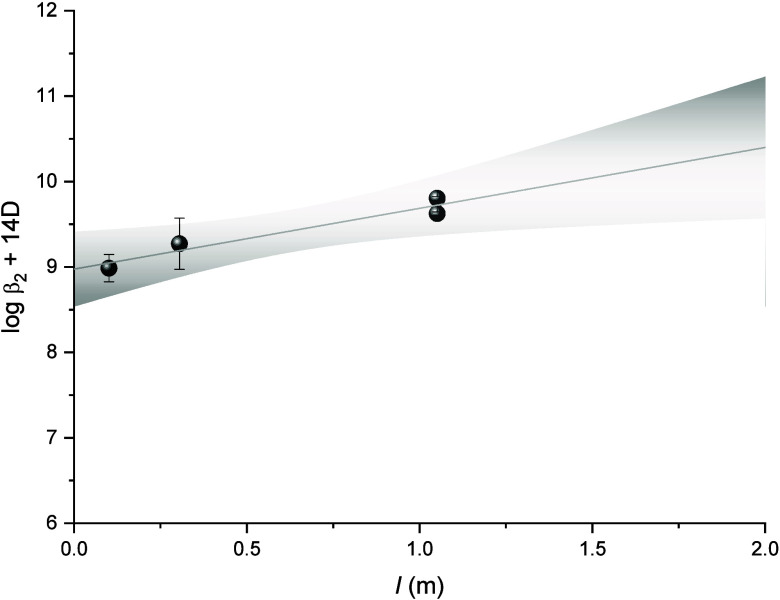
Extrapolation to *I* = 0 of experimental data for the formation of [Th­(AcO)_2_]^2+^ using the specific interaction equation. The data are taken from references 7,9,10 and this study.

### 3.8 Formation Constant of [Th­(AcO)_3_]^+^

Under the conditions of our experiments at *I* = 0.1 M and *I* = 0.3 M NaClO_4_, the acetate concentration was sufficiently high to from the [Th­(AcO)_3_]^+^ complex at pH values where Th­(OH)^3+^ is not relevant. (see Figure S1, Supporting Information). The same is true for previous experiments, meaning the determination of the [Th­(AcO)_3_]^+^ stability constant in literature is not biased and could be compared to the constants determined in the present work. Unfortunately, as depicted in Figure 7, the data of previous studies^7–9^ and this work are scattered which complicates the treatment. We can consider that ε_[Th(AcO)_3_]^+^,ClO_4_^–^_ is reasonably between 0 and ε_[Th(AcO)_2_]^2+^,ClO_4_^–^_. Indeed, according to the determination of logβ_1_^0^ and logβ_2_^0^, the interaction coefficient of ε_[Th(AcO)_3_]^+^,ClO_4_^–^_ is probably lower than ε_[Th(AcO)_2_]^2+^,ClO_4_^–^_ = 0.15 kg mol^–1^ and positive. Such assumptions drastically frame the possible value for logβ_3_^0^, i.e., 11.74 (slope –0.94 kg mol^–1^, ε_[Th(AcO)_3_]^+^,ClO_4_^–^_ = 0, physically impossible but considered here as a limiting value) to 11.85 (ε_[Th(AcO)_3_]^+^,ClO_4_^–^_ = ε_[Th(AcO)_2_]^2+^,ClO_4_^–^_). In both cases, the uncertainty is about 0.5. Therefore, we propose an average value: logβ_3_^0^ = 11.8 ± 0.5.

**Figure 7:**
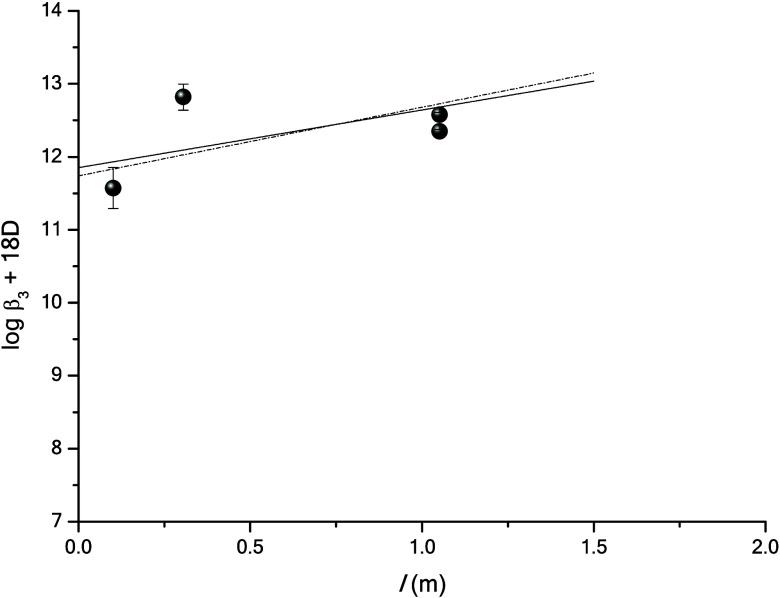
Extrapolation to *I* = 0 of experimental data for the formation of [Th­(AcO)_3_]^+^ using the specific interaction equation. The data are taken from references 7,9,10 and this study. The scattering between data leads to an unphysical value for ε_[Th(AcO)_3_]^+^,ClO_4_^–^_. By considering that 0 < ε_[Th(AcO)_3_]^+^,ClO_4_^–^_ < ε_[Th(AcO)_2_]^2+^,ClO_4_^–^_, the Δε can only vary from −0.94 kg mol^–1^ (straight line) to −0.79 kg mol^–1^ (dotted line).

### 3.9 Formation Constants of [Th­(AcO)_4_] and [Th­(AcO)_5_]^−^

The discovery of stable [Th­(OH)­(AcO)*_i_*]^3–*i*^ species with a behavior independent of the [Th­(AcO)*_i_*]^4–*i*^ species requires us to reconsider all determinations of acetate complexes with four and five acetate ligands. The formation of these complexes occurs in a pH range where the first monohydroxo complex Th­(OH)^3+^ is formed. Indeed, above pH 3, Th­(OH)^3+^ is certainly present, whatever the ionic strength conditions. However, as discussed previously, both types of species [Th­(AcO)*_i_*]^4–*i*^ and [Th­(OH)­(AcO)*_i_*]^3–*i*^ can be separated by CE-ICP-MS. Since the concentration of acetic acid is significantly higher than the concentration of thorium (up to 10^6^ times higher), both bands of migration are surrounded by the same and constant concentration of AcO^–^. Therefore, we can treat both species independently. It is important to understand that CE-ICP-MS is a direct speciation technique unlike potentiometry or solvent extraction, for example.

As a result, former potentiometric determinations of the formation constants of complexes with more than four acetate ligands are biased. However, this is not the case for the 1:1 to 1:3 complexes (see Figure S1 (right), Supporting Information), for which it has been possible to compare the formation constants obtained by several techniques. We have performed CE-ICP-MS experiments at two ionic strengths; at *I* = 0.1 M only one peak has been observed (Figure S5, Supporting Information), whereas at *I* = 0.3 M, the two species [Th­(AcO)*_i_*]^4–*i*^ and [Th­(OH)­(AcO)*_i_*]^3–*i*^ are separated sufficiently to be able to quantify their relative concentration (Figure S4, Supporting Information). Unfortunately, we were not able to detect both species for experiments carried out at *I* = 0.1 M. Due to the lower maximum pH value (pH 3.63), the expected proportion of the [Th­(OH)­(AcO)*_i_*]^3–*i*^ species is smaller compared to the experiments at *I* = 0.3 M. Furthermore, separation condition at *I* = 0.1 M could have not been ideal to allow for the separation of the two species [Th­(AcO)*_i_*]^4–*i*^ and [Th­(OH)­(AcO)*_i_*]^3–*i*^.

For the formation of [Th­(AcO)_4_], our two determinations are a little scattered. We followed the NEA procedure^17^ to cover both determinations at 0.1 and 0.3 M. The mean value of both complex formation constants extrapolated to zero ionic strength was calculated and the uncertainty was selected to cover all data. As a result, the following value is proposed (see Table 3): log_10_β_4_^0^ = 13.9 ± 2.0.

### 3.10 Determination of Complexation Constants for the Th–OH-AcO System

The electrophoretic mobilities assigned to the Th–OH-AcO system under previous considerations (Figure 3, gray symbols) also show a trend with increasing acetate concentration. For the formation of the hydrolyzed acetate complexes, the following equilibrium (log*β_i_) was assumed:

$${\mathrm{Th}}^{4+}+H_{2}O+i{\mathrm{AcO}}^{-}⇌{\left[\mathrm{Th}\left(\mathrm{OH}\right){\left(\mathrm{AcO}\right)}_{i}\right]}^{3-i}+H^{+}$$ 10

This equilibrium consists of the hydrolyzation of Th­(IV) as well as the acetate association. Both formation constants of the individual reactions were determined in the previous sections. To obtain log*β_i_, logβ_*i*_ for the binary acetate complexes and log*K can be combined based on eq 11.

β*=[Th(OH)(AcO)i]3−i]×[H+][Th4+]×[AcO−]i=K*×β=[Th(OH)(AcO)i]3−i]×[H+][Th(AcO)i]4−i×[Th(AcO)i]4−i][Th4+]×[AcO−]i 11

Using the constants determined at *I* = 0.3 M in Table 1 and log*K_OH,AcO_^*I*=0.3M^ = −3.64 ± 0.09, log*β_*i*_^*I*=0.3M^ was calculated. The same was done for the proposed values at *I* = 0 in Table 3 and log*K_OH,AcO_^0^ = −2.7 ± 0.2 to obtain log*β_*i*_^0^. The constants are summarized in Table 4.

**Table 4:** Stability Constants at *I* = 0.3 M and *I* = 0 for the Th-OH-AcO System in NaClO_4_ Medium at 25 °C, as well as Electrophoretic Mobility Values for the Species for the Th-OH-AcO System Determined in This Study

species	μ (10^–8^ m^2^V^–1^s^–1^) *I* = 0.3 M	log*β_*i*_^*I=*0.3M^	log*β_*i*_^0^
[**Th**(**OH**)]**^3*+*^**	+3.243	-	-
[**Th**(**OH**)(**AcO**)]**^2*+*^**	+1.670	0.36 ± 0.25	2.36 ± 0.23
[**Th**(**OH**)(**AcO**)**_2_**]**^*+*^**	+0.943	3.48 ± 0.31	6.28 ± 0.28
[**Th**(**OH**)(**AcO**)**_3_**]	0	6.46 ± 0.24	9.1 ± 0.5
[**Th**(**OH**)(**AcO**)**_4_**]**^*–*^**	–1.150	8.26 ± 0.61	11.2 ± 2.0

Combining all stability constants determined in this work at *I* = 0.3 M for the Th-AcO (Table 1) and Th-OH-AcO (Table 4) systems, the repartition diagram in Figure 8 was calculated.

**Figure 8:**
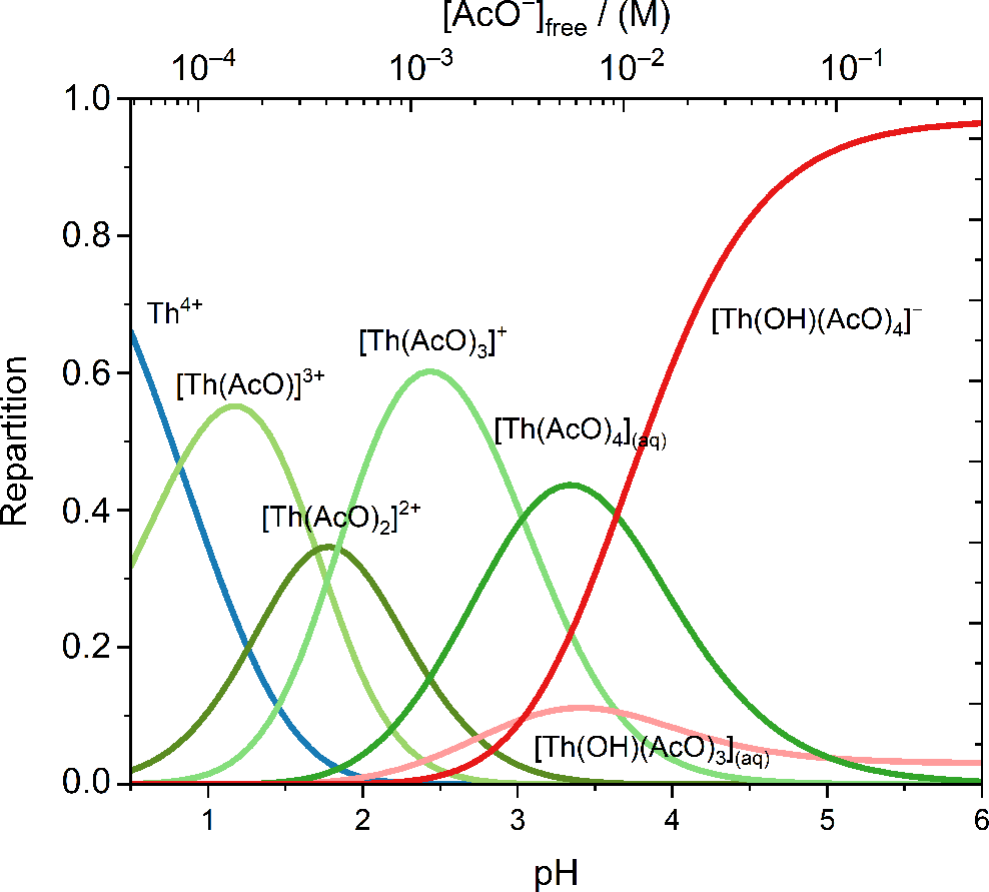
Repartition diagram calculated from all stability constants determined in this work at *I* = 0.3 M NaClO_4_, [AcOH] = 0.5 M and 25 °C (Tables 1 and 4).

The electrophoretic mobilities μ_*eff*_ relative to the Th-OH-AcO system as a function of the concentration of free acetate [AcO^–^]_free_ are shown in Figure 9. To check for the continuity of the data, the trend in electrophoretic mobility expected for the Th-OH-Ac system was calculated based on the speciation plotted in Figure 8. The individual mobilities of the Th-OH-Ac complexes were selected based on the Th-Ac complexes of the same charge (Table 2). For the [Th­(OH)­(AcO)_4_]^−^ complex, an electrophoretic mobility of −1.15 × 10^–8^ m^2^V^–1^s^–1^ was estimated. All mobilities are summarized in Table 4.

**Figure 9:**
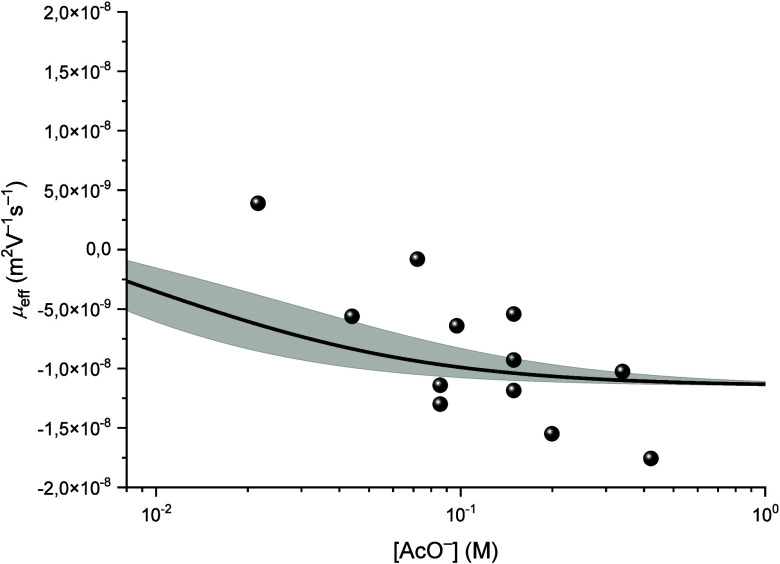
Variation of electrophoretic mobility μ_eff_ of the [Th­(OH)­(AcO)*_i_*]^3–*i*^ species from this work and retreated from Willberger et al.^10^ as a function of the concentration of free acetate [AcO^–^]_free_ at *I* = 0.3 M. The expected trend in electrophoretic mobility based on the Th speciation shown in Figure 8 is shown as a black line with the uncertainties in gray.

The electrophoretic mobility values assigned to the Th-OH-AcO system scattered significantly more compared to the Th-AcO system. We observed that hydrolyzed species systematically led to broad, distorted peaks, evidence of the presence of other species (most likely hydroxides) (Figure S4, Supporting Information). It is also possible that this may be accompanied by interaction with the fused silica capillary, further degrading the resolution. The peak is therefore no longer associated with a single species, which explains the observed discrepancies. All things considered, the experimental data mostly scatters around the expected trend, which can be seen as confirmation of the continuity of the proposed formation constants.

## 4 Conclusion

The Th/acetate system is more complex than expected. We have detected the formation of [Th­(OH)­(AcO)*_i_*]^3–*i*^ species, that question the reliability of the literature. These species were detected independently, with two different CE-ICP-MS systems, by Willberger et al.^10^ in 2019 and presently in this study. The domain of existence of these [Th­(OH)­(AcO)*_i_*]^3–*i*^ species is in competition with the formation of the [Th­(AcO)_5_]^−^ complex. Previous studies have therefore underestimated the formation constant of the 1:4 complex, while the constant associated with the 1:5 complex is in fact an overall constant associated with the formation of [Th­(OH)­(AcO)*_i_*]^3–*i*^. However, the formation constants of the 1:1 to 1:3 complexes are not affected by the presence of the hydrolyzed species. We have therefore used literature values and SIT theory to determine the formation constants extrapolated at zero ionic strength. All values are mutually consistent. This system is truly original in that it combines two types of complexes: stable and labile. This is the first time we have encountered this type of chemical system. Under the experimental conditions, only the first hydrolysis was detected. The study of peak area variations for kinetically stable complexes is only dependent on pH. This has enabled us to determine the first formation constant of Th­(OH)^3+^, a more precise value, in excellent agreement with that recommended in the NEA review done by Rand et al.^14^ The study of variations in electrophoretic mobilities for labile complexes showed that the two species Th^4+^ and Th­(OH)^3+^ were independently complexed by acetate ligands to form the species [Th­(AcO)*_i_*]^4–*i*^ (*i* = 1 – 4) and [Th­(OH)­(AcO)*_j_*]^3–*j*^ (*j* = 1 – 4). The next article will be devoted to the acetate complexation of plutonium­(IV), which is also full of surprises.

## Supplementary Material



## Notes

Speciation diagrams, experimental parameters, and electropherograms (PDF)
