# Supplementary material for: The Th-Acetate Chemical Equilibria: Is It Really That Simple?
Source: Inorg Chem. 2025 Nov 10;64(46):22674–82. doi: 10.1021/acs.inorgchem.5c03418 (PMC12648665; doi:10.1021/acs.inorgchem.5c03418)
Supplement: Supplementary file 1 [file ic5c03418_si_001.pdf]

SUPPORTING INFORMATION

The Th-Acetate Chemical Equilibria: Is It Really That Simple?

*Janik Lohmann[a], Christelle Tamain[b], Philippe Moisy[b], Tobias Reich[a] *, and Jean Aupiais[c] **

[a] Johannes Gutenberg-Universität Mainz, Department of Chemistry - Nuclear Chemistry, 55099 Mainz, Germany.

[b] CEA, DES, ISEC, DMRC, Univ Montpellier, F-30207 Marcoule, France

[c] CEA, DAM, DIF, F-91297 Arpajon Cedex, France.

* Corresponding authors: treich@uni-mainz.de, jean.aupiais@cea.fr

The Supporting Information comprises 16 pages,

including 5 figures, 7 tables, and 3 references.

Contents

Additional data 2
Sample preparation 3
Sample preparation in 0.3 M ionic strength 3
Electrolyte preparation in 0.3 M ionic strength 4
Sample preparation in 0.1 M ionic strength 5
Experimental results 6
Experimental results at 0.3 M ionic strength 6
Experimental results at 0.1 M ionic strength 7
pH measurements in 0.3 M NaClO4 8
Electropherograms 9
Electropherograms at 0.3 M ionic strength 9
Electropherograms at 0.1 M ionic strength 14
References 16

Additional data

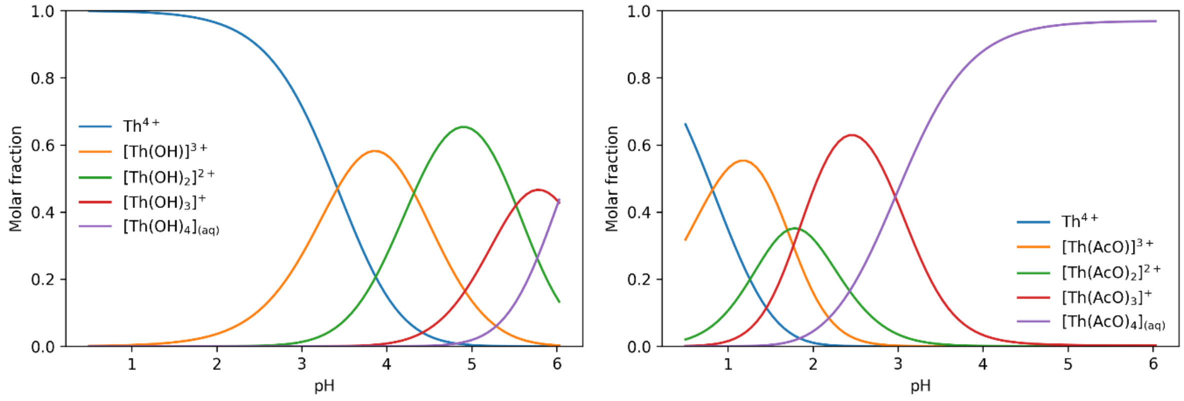

Figure S1. Speciation diagrams for Th(IV) (left) for the hydrolysis, calculated manually using the NEA[1] recommended values extrapolated using SIT[2] and (right) for the Th-Ac system for [AcOH] = 0.5 M calculated manually using the constants recommended in this work (Table 1-main), both at I = 0.3 M.

Table S1. Specific ion interaction coefficients in $NaClO_4$ medium.[1]

Coefficient (kg mol^{-1})	Value
$\varepsilon_{Th^{4+},ClO_4^-}$	0.70 ± 0.10
$\varepsilon_{Th^{4+},Cl^-}$	0.25 ± 0.03
$\varepsilon_{Th^{4+},NO_3^-}$	0.31 ± 0.12
ε_{AcO^-,Na^+}	0.08 ± 0.01
ε_{AcO^-,K^+}	0.09 ± 0.01
$\varepsilon_{[Th(AcO)]^{3+},ClO_4^-}$	$0.39 \pm 0.17^{\#}$
$\varepsilon_{[Th(AcO)]^{3+},Cl^-}$	$0.17 \pm 0.10^*$
$\varepsilon_{[Th(AcO)]^{3+},NO_3^-}$	$0.20 \pm 0.10^*$
$\varepsilon_{[Th(AcO)_2]^{2+},ClO_4^-}$	$0.15 \pm 0.10^{\#}$
$\varepsilon_{[Th(AcO)_2]^{2+},Cl^-}$	$0.14 \pm 0.10^*$
$\varepsilon_{[Th(AcO)_2]^{2+},NO_3^-}$	$0.16 \pm 0.10^*$
$\varepsilon_{[Th(AcO)_3]^{+},ClO_4^-}$	$0.24 \pm 0.10^*$
$\varepsilon_{[Th(AcO)_4],ClO_4^-}$	0

* estimated by Caviatta's method.[3], # experimental value, this study

Sample preparation

Sample preparation in 0.3 M ionic strength

Remark: Pu^{4+} has been introduced with Th^{4+} for comparison. Data relative to the complexation of Pu^{4+} by acetate anion will be published later.

Table S2. Sample composition at I = 0.3 M.

Sample #	V_{total} (µL)	V_{Pu} (µL)	V_{Th} (µL)	V_{NaOH} (µL)	V_{HClO4} (µL)	V_{AcOH} (µL)	V_{NaClO4} (µL)	V_{water} (µL)	Voltage (kV)
1	100	5	5	0	30	45	0	15.0	+4
2	100	5	5	0	15	45	7.5	22.5	+4
3	100	5	5	0	7.5	45	11.2	26.3	+4
4	100	5	5	0	2	45	14.0	29.0	+4
5	100	5	5	0	**10**	45	14.5	20.5	+7
6	100	5	5	0	0	45	14.8	30.2	+7
7	100	5	5	**5**	0	45	14.7	25.3	+7
8	100	5	5	**10**	0	45	14.5	20.5	+7
9	100	5	5	2.5	0	45	13.7	28.8	–7 ; +7
10	100	5	5	5	0	45	12.5	27.5	–7 ; +7
11	100	5	5	7.5	0	45	11.2	26.3	–7 ; +7
12	100	5	5	10	0	45	10.0	25.0	–7
13	100	5	5	20	0	45	5.0	20.0	–7
14	100	5	5	30	0	45	0	15.0	–7
15	100	5	5	0	4.3	45	12.8	27.8	+4
16	100	5	5	0	2.7	45	13.6	28.6	+4
17	100	5	5	0	1.4	45	14.3	29.3	+7
18	100	5	5	0	0.5	45	14.7	29.7	+7
19	100	5	5	0	**2**	45	14.9	28.1	+7
20	100	5	5	1.2	0	45	14.4	29.4	–7 ; +7
21	100	**5**	**5**	0	27	10	0	53.0	+4
22	100	**5**	**5**	0	27	5	0	58.0	+4
23	100	**5**	**5**	0	27	1	0	62.0	+4
24	100	**5**	**5**	0	27	0.5	0	62.5	+4
25	100	**5**	**5**	0	27	0.2	0	62.8	+4
26	100	**5**	**5**	0	27	0	0	63.0	+4

Pu stock solution: $C_{Pu} = 10^{-7}$ M in 0.5 M acetic acid, or $C_{Pu} = 10^{-7}$ M in 0.3 M $HClO_4$ (in pink)

Th stock solution: $C_{Th} = 2 \times 10^{-6}$ M in 0.5 M acetic acid, or $C_{Th} = 2 \times 10^{-6}$ M in 0.3 M $HClO_4$ (in pink)

NaOH stock solution: C = 1 M or 0.1 M **(in red)**.

$HClO_4$ stock solution: C = 1 M or 0.1 M **(in blue)**.

$NaClO_4$ stock solution: C = 2 M.

AcOH acetic acid stock solution: C = 1 M.

Electrolyte preparation in 0.3 M ionic strength

Table S3. Electrolyte composition at *I* = 0.3 M.

Sample #	p_cH	$[AcO^-]$ (M)	V_{total} (μL)	V_{NaOH} (μL)	V_{HClO4} (μL)	V_{AcOH} (μL)	V_{NaClO4} (μL)	V_{water} (μL)
1	0.99	1.484×10^{-4}	1000	0	300	500	0	200.0
2	1.20	2.402×10^{-4}	1000	0	150	500	75.0	275.0
3	1.39	3.735×10^{-4}	1000	0	75	500	112.4	312.6
4	1.97	1.403×10^{-4}	1000	0	20	500	139.7	340.3
5	2.30	2.999×10^{-3}	1000	0	10	500	144.5	345.5
6	2.70	7.483×10^{-3}	1000	0	0	500	148.4	351.6
7	2.94	1.298×10^{-2}	1000	5	0	500	146.7	348.3
8	3.10	1.843×10^{-2}	1000	10	0	500	144.5	345.5
9	3.50	4.395×10^{-2}	1000	25	0	500	137.3	337.7
10	3.83	8.564×10^{-2}	1000	50	0	500	124.9	325.1
11	4.15	1.493×10^{-1}	1000	75	0	500	112.4	312.6
12	4.34	1.992×10^{-1}	1000	100	0	500	99.9	300.1
13	4.84	3.399×10^{-1}	1000	200	0	500	50.0	250.0
14	5.25	4.212×10^{-1}	1000	300	0	500	0	200.0
15	1.71	7.690×10^{-4}	1000	0	43.1	500	128.5	328.5
16	1.84	1.060×10^{-3}	1000	0	27.1	500	136.5	336.5
17	2.07	1.783×10^{-3}	1000	0	14.2	500	142.9	342.9
18	2.33	3.248×10^{-3}	1000	0	5.1	500	147.4	347.4
19	2.56	5.450×10^{-3}	1000	0	2.0	500	149.0	349.0
20	3.20	2.321×10^{-2}	1000	12	0.0	500	144.0	344.0
21*	1.09	1.013×10^{-5}	1000	0	300	100	0	600.0
22*	1.01	5.070×10^{-6}	1000	0	300	50	0	650.0
23*	0.96	1.010×10^{-6}	1000	0	300	10	0	690.0
24*	0.99	5.070×10^{-7}	1000	0	300	5	0	695.0
25*	1.04	2.027×10^{-7}	1000	0	300	2	0	698.0
26*	1.01	0.000	1000	0	300	0	0	700.0

$[AcO^-]$ calculated from pKa of acetic acid, at this acidity, the concentration of proton is controlled by $HClO_4$ and not by AcOH.

Sample preparation in 0.1 M ionic strength

NaOH stock solution: C = 10 M.
$HClO_4$ stock solution: C = 9 M.

Table S4. Sample composition at *I* = 0.1 M.

Sample #	V_{tot} (mL)	pH	[AcOH] (M)	[AcO^-] (M)	V_{HClO4} (μL)	V_{NaOH} (μL)	m_{NaClO4} (g)
1	10.09	0.70	0.75	1.04×10^{-4}	90	0	0
2	10.05	1.09	0.75	2.55×10^{-4}	45	0	0.0794
3	10.03	1.27	0.75	3.86×10^{-4}	25	0	0.1071
4	10.02	1.57	0.75	7.70×10^{-4}	15	0	0.1208
5	10.01	1.68	0.75	9.92×10^{-4}	10	0	0.1274
6	10.00	2.03	0.75	2.22×10^{-3}	0	0	0.1338
7	10.01	2.41	0.75	5.30×10^{-3}	0	5	0.1271
8	10.02	2.62	0.75	8.55×10^{-3}	0	15	0.1148
9	10.02	2.79	0.75	1.26×10^{-2}	0	20	0.1076
10	10.03	3.02	0.75	2.11×10^{-2}	0	30	0.0901
11	10.04	3.16	0.75	2.88×10^{-2}	0	40	0.0754
12	10.08	3.49	0.75	5.91×10^{-2}	0	75	0.0270
13	10.10	3.63	0.75	7.92×10^{-2}	0	100	0
14	10.05	1.36	0.75	4.75×10^{-4}	45	0	0.0793
15*	10.00	1.33	0.075	4.43×10^{-5}			
16*	10.00	1.33	0.025	1.48×10^{-5}			
17*	10.00	1.33	0.0075	4.43×10^{-6}			
18*	10.00	1.35	0.0025	1.55×10^{-6}			

* Samples were produced by diluting sample 14 in a solution of 0.05 M $NaClO_4$ and 0.05 M $HClO_4$ accordingly.

Pu stock solution: $C_{Pu} = 2 \times 10^{-4}$ M in 1 M $HClO_4$.
Th stock solution: $C_{Th} = 2 \times 10^{-4}$ M in 0.1 M $HClO_4$.
Cs stock solution: $C_{CsCl} = 1 \times 10^{-3}$ M in H_2O.

To 2 mL of the samples BGE 2 μL of each stock were added. No significant change in pH was observed upon addition. The pH values of the samples were measured immediately before the CE-ICP-MS measurement.

Experimental results

Experimental results at 0.3 M ionic strength

Table S5. Migration times and electrophoretic mobility of Th for each sample at $I = 0.3$ M.

Sample #	Measured pH	Free [AcO⁻] (M)	Migration time species t_{Th} (s)	Migration time EOF t_{EOF} (s)	Electrophoretic mobility μ (10^{-8} $m^2V^{-1}s^{-1}$)
1	0.99	1.484×10^{-4}	471.772	530.777	3.538
2	1.20	2.402×10^{-4}	490.595	541.443	2.874
3	1.39	3.735×10^{-4}	498.127	547.226	2.705
4	1.97	1.403×10^{-4}	*535.023*	*552.173*	*0.872*
5	2.30	2.999×10^{-3}	517.217	556.746	1.178
6	2.70	7.483×10^{-3}	530.898	554.762	0.695
7	2.94	1.298×10^{-2}	537.317	552.943	0.695
8	3.10	1.843×10^{-2}	540.780	550.071	0.268
9	3.50	4.395×10^{-2}	682.300<br>566.839	680.505<br>546.591	$0.033^{<0}$<br>$-0.331^{>0}$
10	3.83	8.564×10^{-2}	623.794<br>591.941	688.865<br>548.728	$-1.300^{<0}$<br>$-1.142^{>0}$
11	4.15	1.493×10^{-1}	*494.969*<br>668.999<br>*593.859*	*522.957*<br>698.502<br>*548.877*	*$-0.928^{<0}$*<br>$-0.542^{<0}$<br>*$-1.184^{>0}$*
12	4.34	1.992×10^{-1}	624.065	703.258	–1.548
13	4.84	3.399×10^{-1}	662.930	719.971	–1.025
14	5.25	4.212×10^{-1}	647.741	746.706	–1.756
15	1.71	7.690×10^{-4}	687.528	754.953	1.951
16	1.84	1.060×10^{-3}	*714.838*	*756.606*	*1.156*
17	2.07	1.783×10^{-3}	672.436	754.059	1.381
18	2.33	3.248×10^{-3}	699.130	754.859	0.906
19	2.56	5.450×10^{-3}	694.043	752.230	0.956
20	3.20	2.321×10^{-2}	*820.523*<br>775.48	*681.240*<br>752.973	*$2.138^{<0}$*<br>$-0.331^{>0}$
21	1.09	1.013×10^{-5}	579.510	705.026	4.613
22	1.01	5.070×10^{-6}	569.592	700.939	4.940
23	0.96	1.010×10^{-6}	564.477	696.360	5.038
24	0.99	5.070×10^{-7}	565.983	695.822	4.950
25	1.04	2.027×10^{-7}	560.240	695.743	5.220
26	1.01	0.000	549.203<br>557.903	691.238<br>693.559	5.618<br>5.264

in grey: rejected, outlier data; in red: hydrolyzed acetate complex; $^{>0}$ positive voltage; $^{<0}$ negative voltage.

Experimental results at 0.1 M ionic strength

Table S6. Migration times and electrophoretic mobility of Th for each sample at I = 0.1 M.

Sample #	Measured pH	Free $[AcO^-]$ (M)	Migration time species t_{Th} (s)	Migration time EOF t_{EOF} (s)	Electrophoretic mobility μ (10^{-8} $m^2V^{-1}s^{-1}$)
1	0.70	1.04×10^{-4}	182.640	263.700	4.208
2	1.09	2.55×10^{-4}	199.560	276.540	3.487
3	1.27	3.86×10^{-4}	220.080	289.860	2.735
4	1.57	7.70×10^{-4}	226.800	300.120	2.693
5	1.68	9.92×10^{-4}	237.540	308.340	2.417
6	2.03	2.22×10^{-3}	247.320	310.920	2.068
7	2.41	5.30×10^{-3}	255.000	310.920	1.763
8	2.62	8.55×10^{-3}	263.700	309.360	1.399
9	2.79	1.26×10^{-2}	263.220	302.700	1.239
10	3.02	2.11×10^{-2}	273.360	304.740	0.942
11	3.16	2.88×10^{-2}	272.940	298.080	0.773
12	3.49	5.91×10^{-2}	278.580	289.380	0.335
13	3.63	7.92×10^{-2}	287.280	291.420	0.124
14	1.36	4.75×10^{-4}	206.040	286.620	3.411
15	1.33	4.43×10^{-5}	173.460	260.820	4.827
16	1.33	1.48×10^{-5}	170.400	258.960	5.017
17	1.33	4.43×10^{-6}	170.400	259.560	5.040
18	1.35	1.55×10^{-6}	174.060	258.360	4.686

in grey: rejected, outlier data

pH measurements in 0.3 M $NaClO_4$

Table S7. $[H^+]$ solutions in 0.3 M $NaClO_4$ prepared for pH electrode calibration and potentials measured.

Sample	$[H^+]$ (M)	V (mV)	pcH
**1**	$2.51\ 10^{-1}$	360.6	0.60
**2**	10^{-1}	342.8	1.00
**3**	10^{-2}	290.7	2.00
**4**	10^{-3}	234.2	3.00
**5**	10^{-4}	171.0	4.00
**6**	10^{-5}	111.6	5.00
**7**	$5.75\ 10^{-8}$	–15.2	7.24

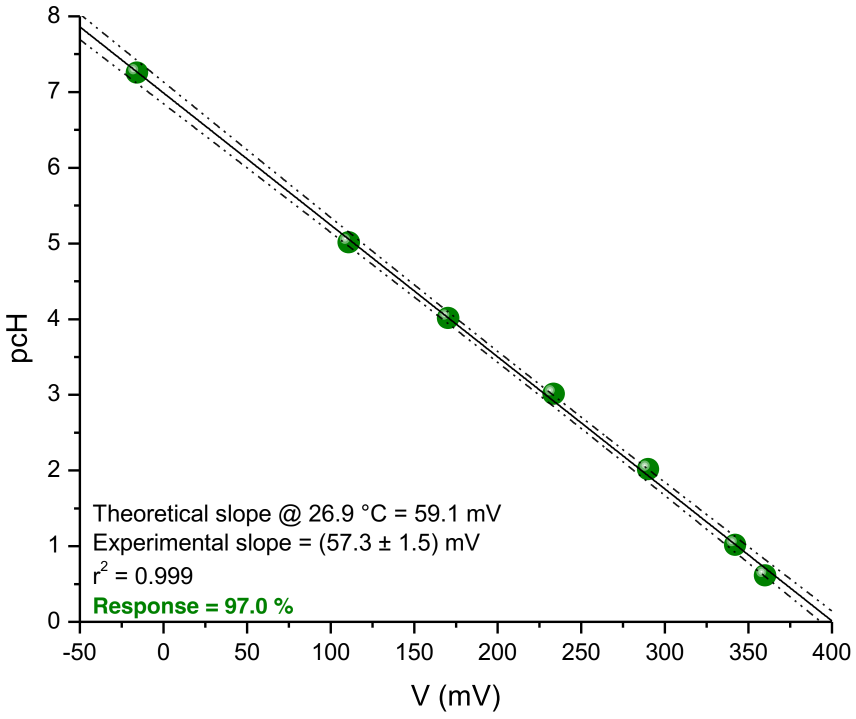

Figure S2. Response of a pH electrode conditioned in 0.3 M $NaClO_4$.

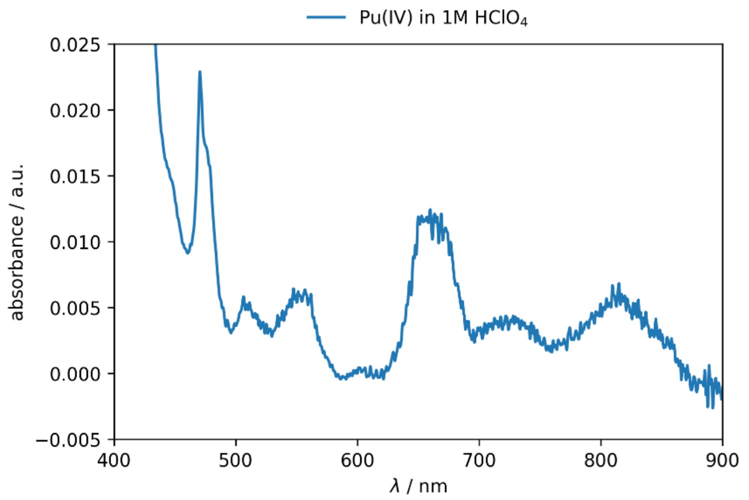

Figure S3. UV-vis spectrum of 2 × 10^{-4} M Pu(IV) in 1 M $HClO_4$.

Electropherograms

The extra features of the electropherograms are caused by external influences, like the artefacts caused by the sample introduction. Internal standards were used to identify these artifacts and rule out that they are actual Th species. The spikes are likely caused by colloidal aggregates on the capillary. We found that as long we still detect a solvated species, colloidal aggregates have no influence.

Electropherograms at 0.3 M ionic strength

Related to Table S5

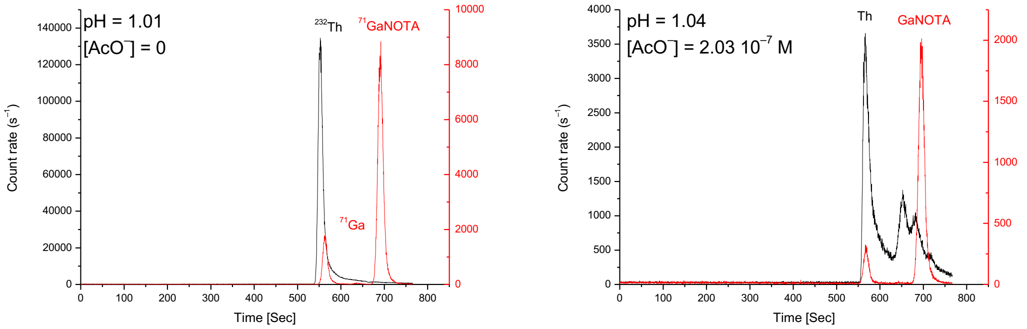

Figure S4. Electropherograms of ^{232}Th and ^{71}Ga (EOF) at 0.3 M ionic strength.

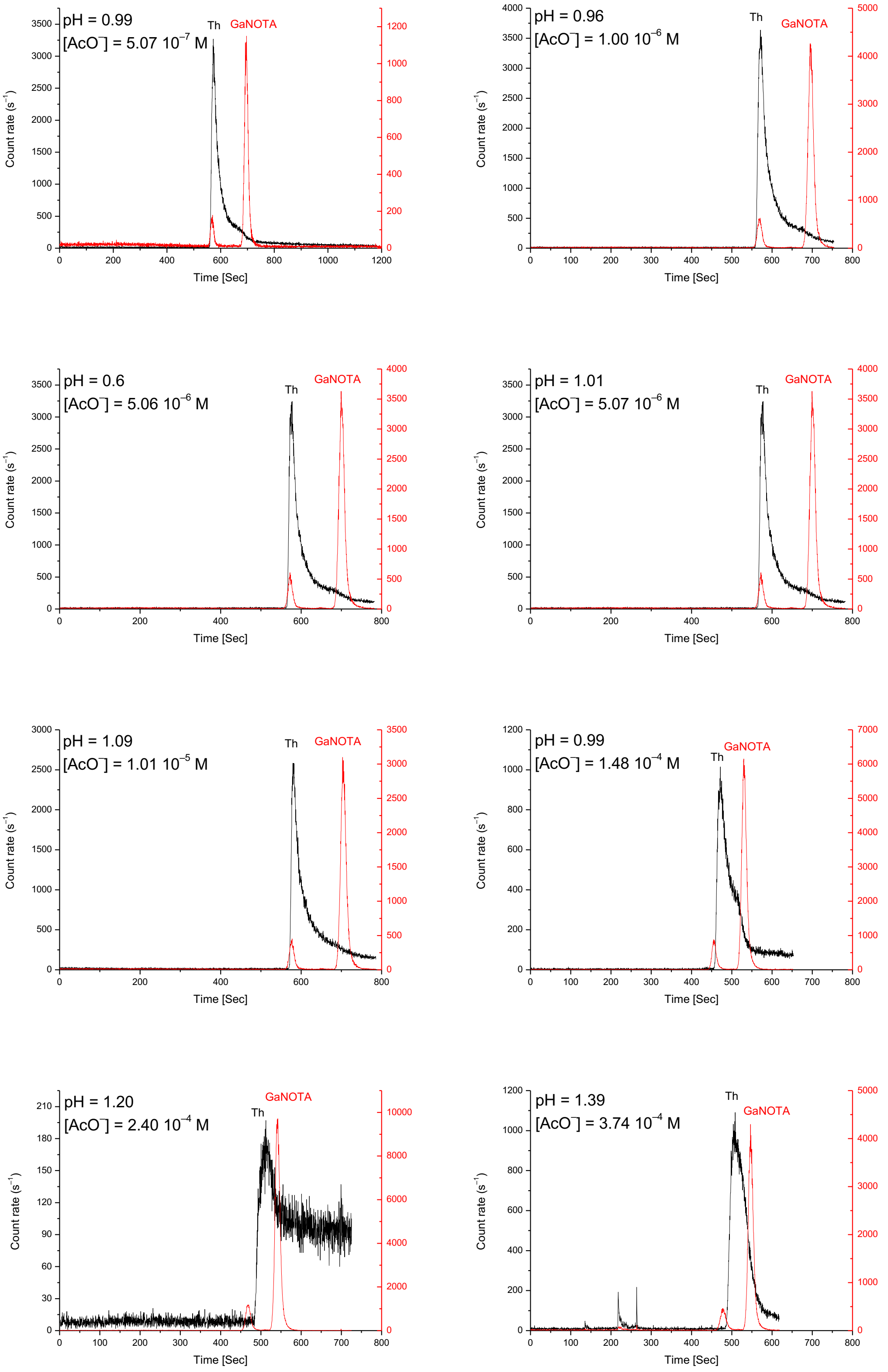

Figure S4. (continued).

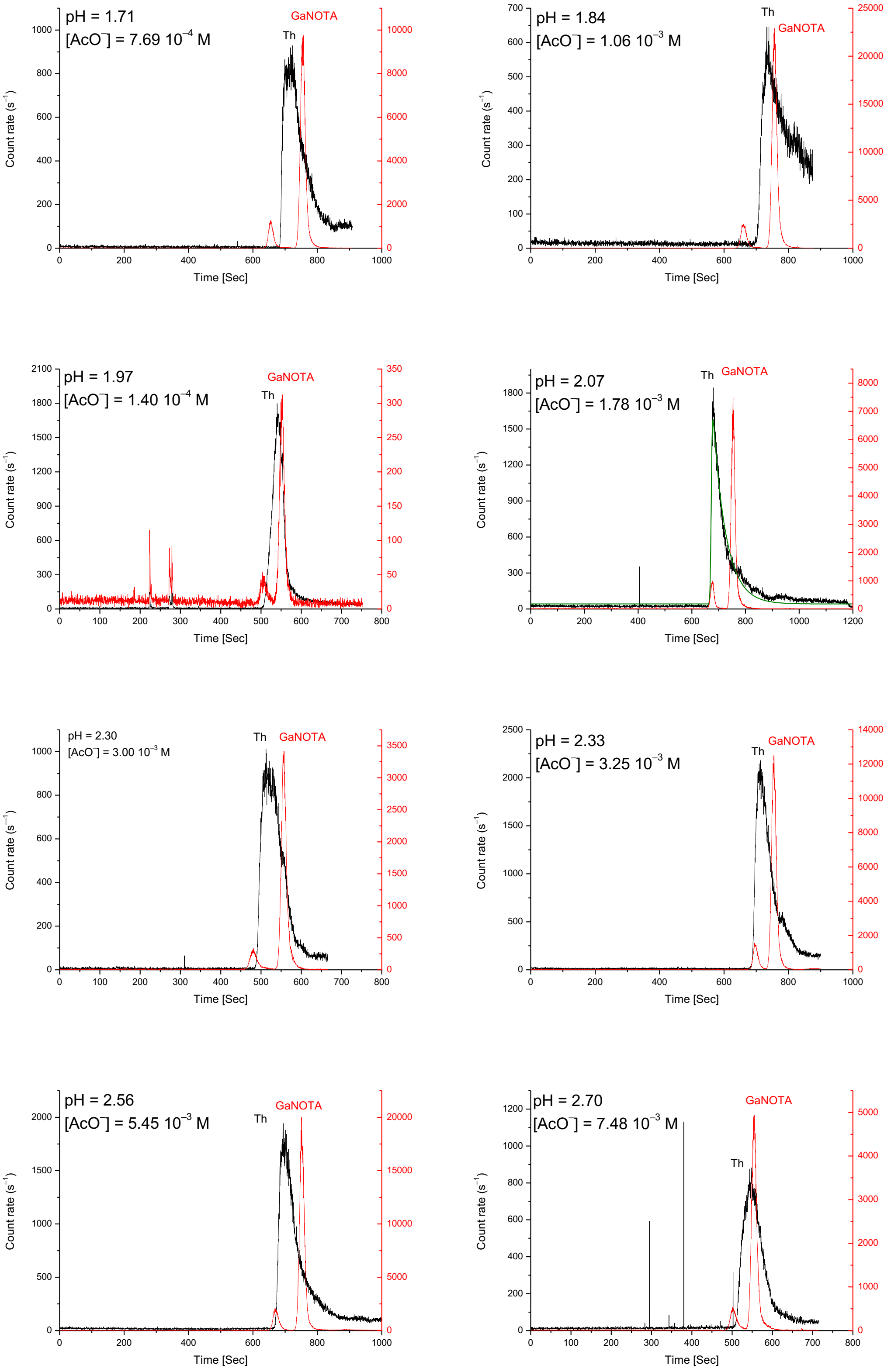

Figure S4. (continued).

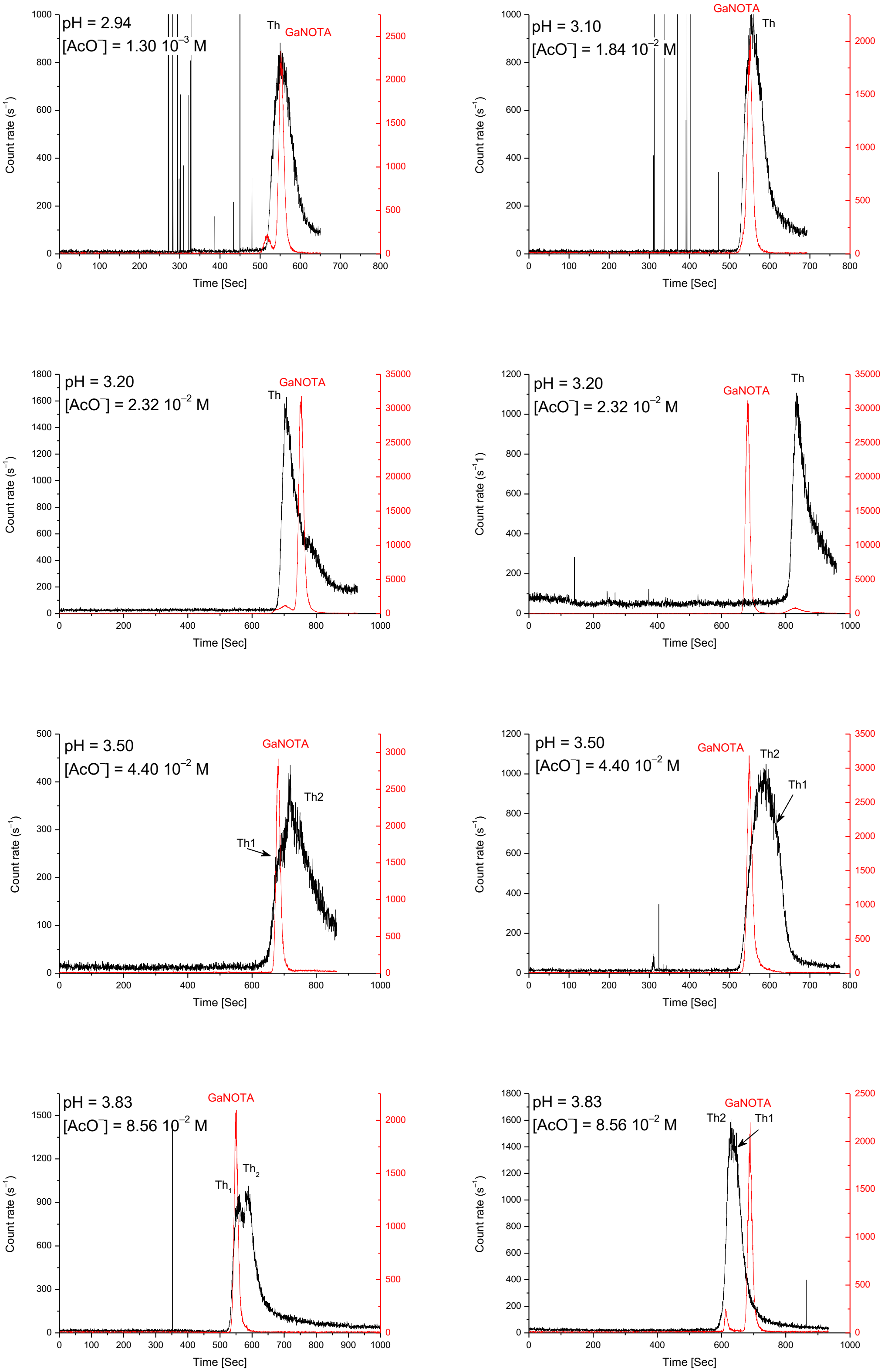

Figure S4. (continued).

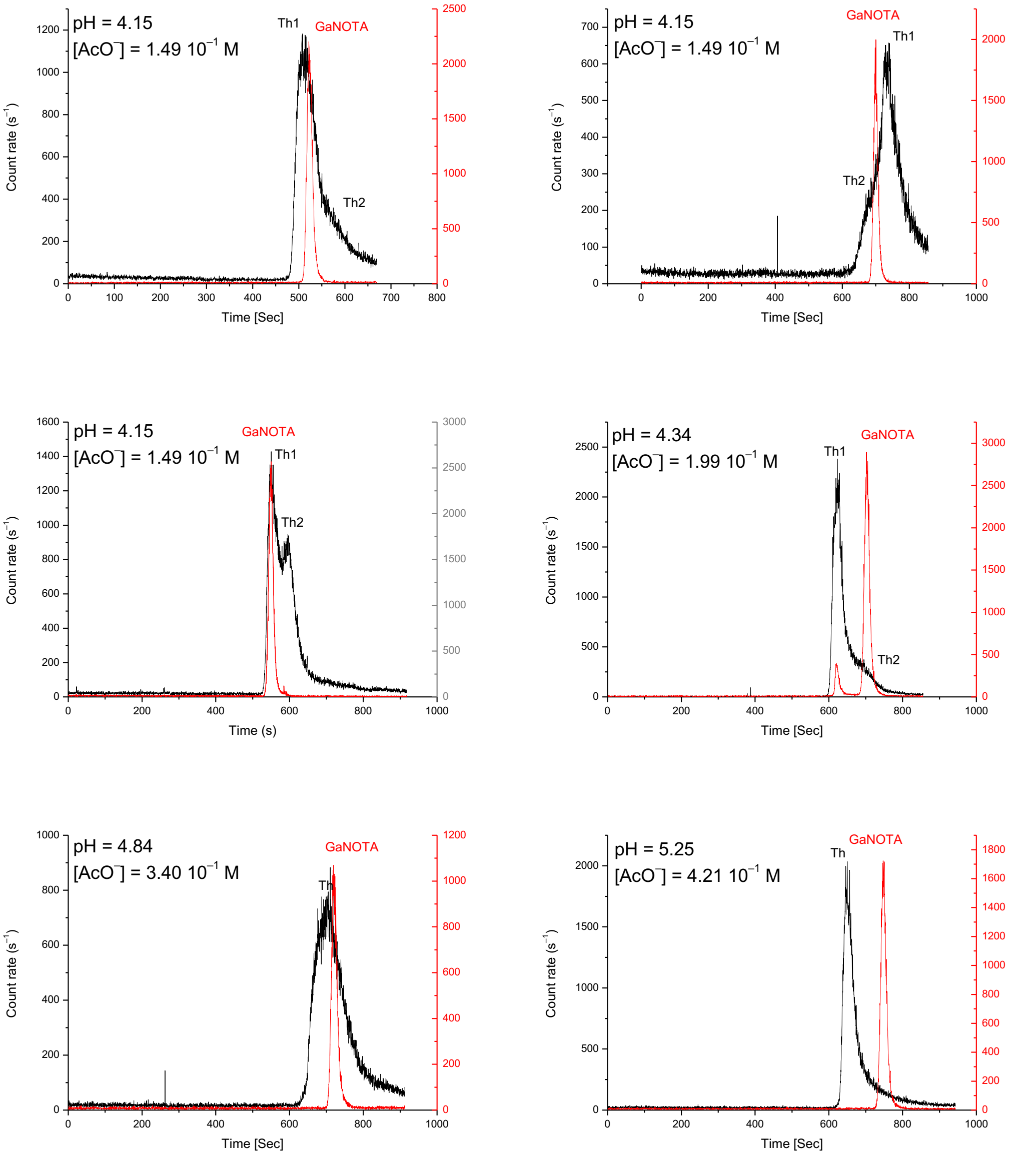

Figure S4. (continued).

Electropherograms at 0.1 M ionic strength

Related to Table S6

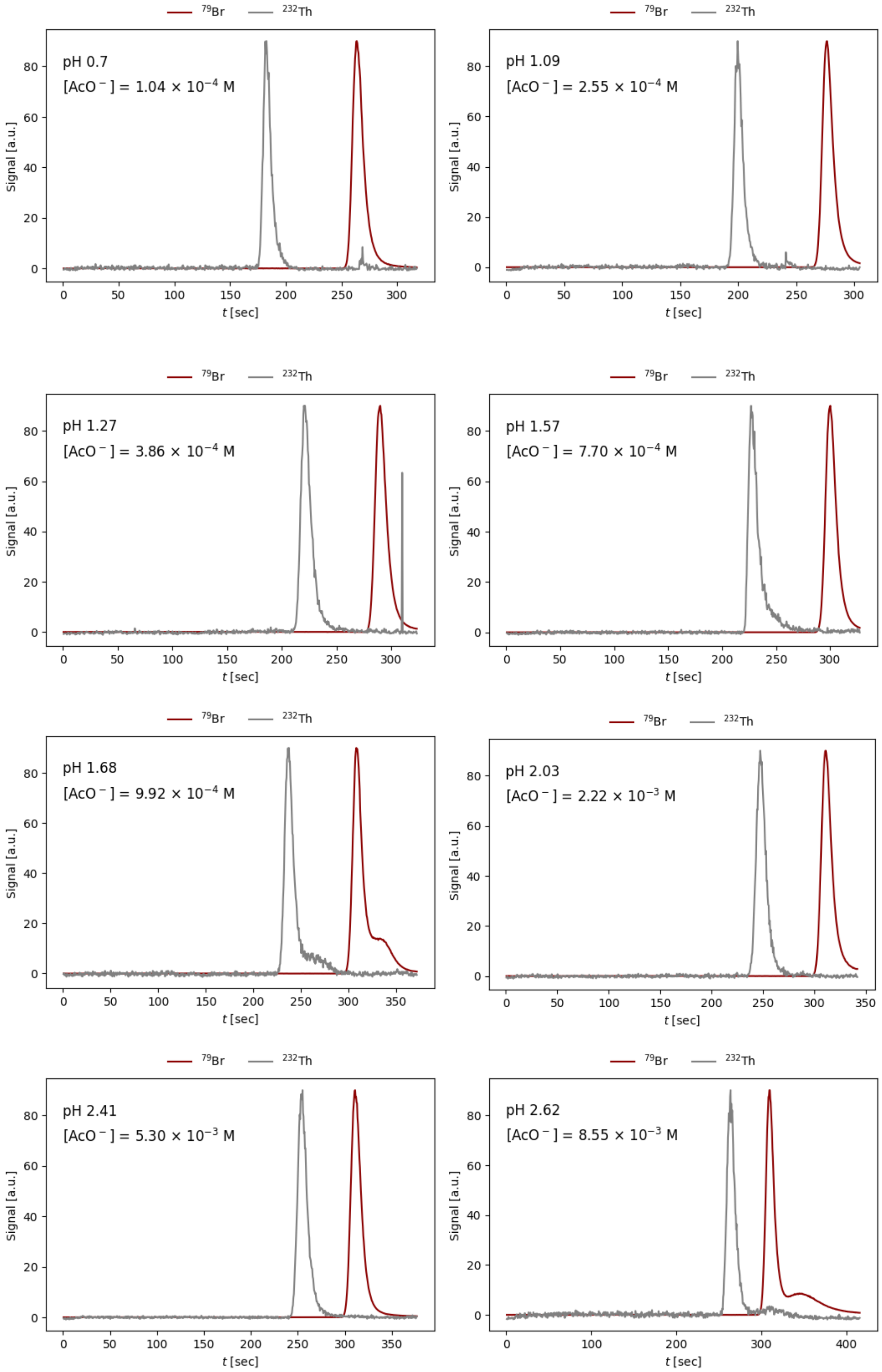

Figure S5. Electropherograms of ^{232}Th and ^{79}Br (EOF) at 0.1 M ionic strength.

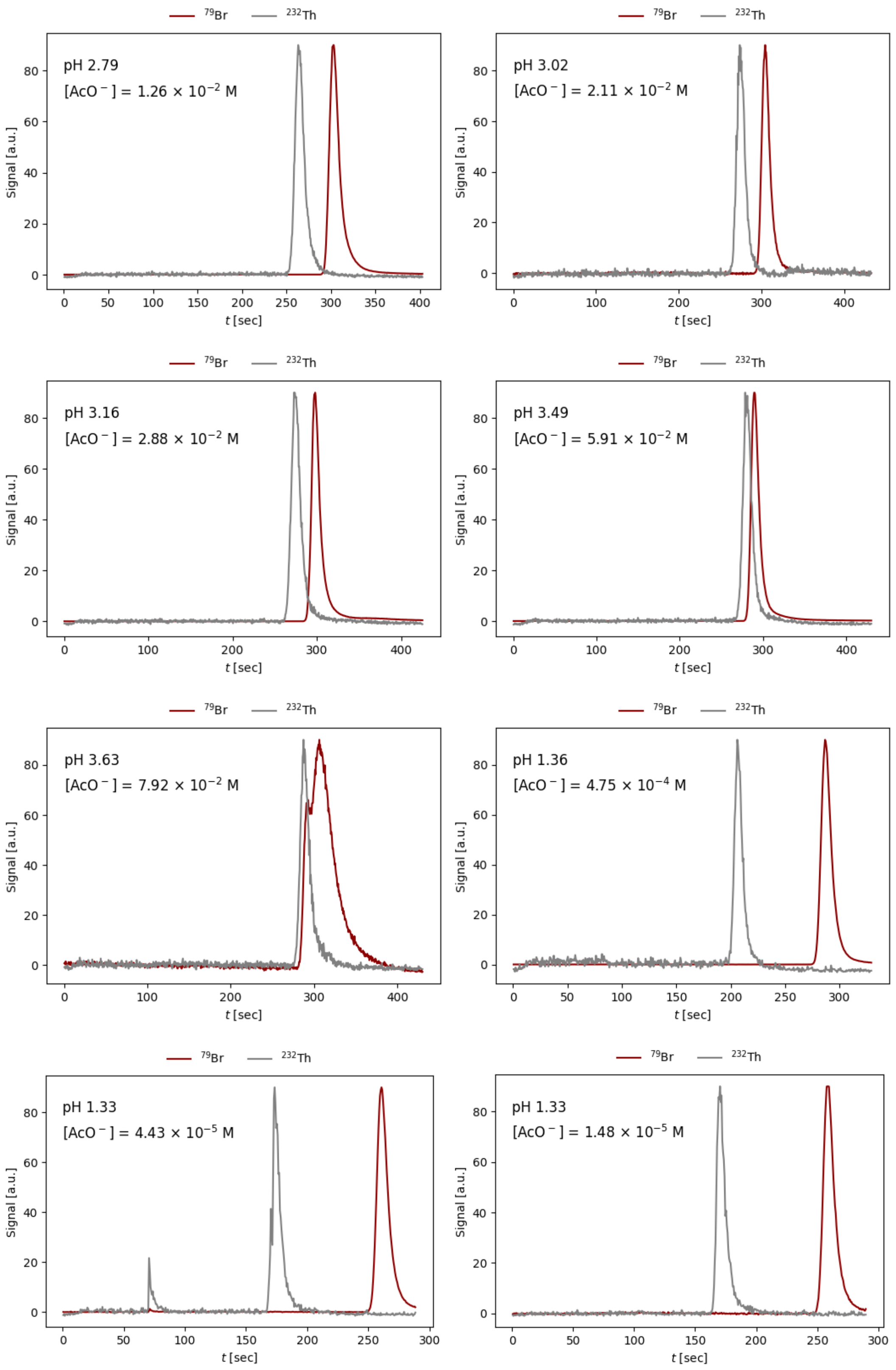

Figure S5. (continued).

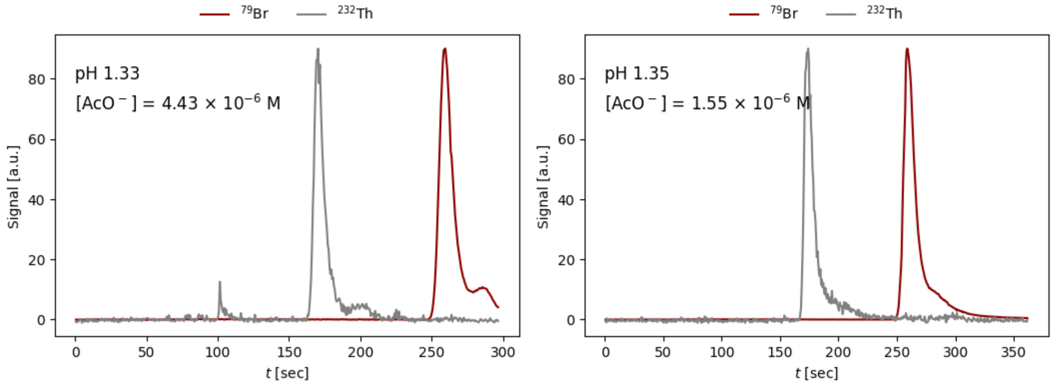

Figure S5. (continued).

References

(1) NEA. *Chemical Thermodynamics of Thorium*; Chemical Thermodynamics, Vol. 11; OECD Publishing, 2009.

(2) NEA. *TDB-2: Guidelines for the Extrapolation to Zero Ionic Strength*; OECD Publishing, 2013.

(3) Ciavatta, L. The specific interaction theory in equilibrium analysis. Some empirical rules for estimating interaction coefficients of metal ion complexes. *Ann. Chim. (Rome)* **1990** (80), 255–263.
